# Supplementary material for: Sounds familiar(?): Expertise with specific musical genres modulates timing perception and micro-level synchronization to auditory stimuli
Source: Atten Percept Psychophys. 2021 Dec 3;84(2):599–615. doi: 10.3758/s13414-021-02393-z (PMC8888399; doi:10.3758/s13414-021-02393-z)
Supplement: Supplementary file 1 — (DOCX 16914 kb) [file 13414_2021_2393_MOESM1_ESM.docx]

**Sounds familiar(?) Expertise with specific musical genres modulates timing perception and micro-level synchronization to auditory stimuli**

Anne Danielsen^ab^, Kristian Nymoen^ac^, Martin Torvik Langerød^ab^, Eirik Jacobsen^ab^,

Mats Johansson^d^, Justin London^e^

^a^RITMO Center for Interdisciplinary Studies of Rhythm, Time, and Motion, University of Oslo, Norway

^b^Department of Musicology, University of Oslo, Norway

^c^Department of Informatics, University of Oslo, Norway

^d^University of South-Eastern Norway

^e^Carleton College, USA

**Abstract**

Musical expertise improves the precision of timing perception and performance—but is this expertise generic, or is it tied to the specific style(s) and genre(s) of one’s musical training? We asked expert musicians from three musical genres (folk, jazz and EDM/hip-hop) to align click tracks and tap in synchrony with genre-specific and genre-neutral sound stimuli to determine the perceptual center (‘P-center’) and variability (‘beat bin’) for each group of experts. We had three stimulus categories –Organic, Electronic and Neutral sounds– each of which had a 2x2 design of the acoustic factors of Attack (fast/slow) and Duration (short/long). We found significant effects of Genre expertise, and a significant interaction for both P-center and P-center variability: folk and jazz musicians synchronize to sounds typical of folk and jazz in a different manner than the EDM/hip-hop producers. The results show that expertise in a specific musical genre affects our low-level perceptions of sounds as well as their affordance(s) for joint action/synchronization. The study provides new insights into the effects of active long-term musical enculturation and skill acquisition on basic sensorimotor synchronization and timing perception, shedding light on the important question of how nature and nurture intersect in the development of our perceptual systems.

*Keywords:* P-center; beat bin; synchronization; timing; tapping; genre; musical expertise

*APP Keywords*: music cognition, sound recognition; psychoacoustics; temporal processing

**Introduction**

When musicians synchronize the sounds they make with those of another musician, that synchrony may be achieved with greater or lesser precision. Increased musical expertise is generally regarded as improving the precision of both timing perception and performance. As yet, however, there is little understanding as to how this expertise is affected by musical enculturation, that is, by specialized forms of musical skill and experience. Musical enculturation can take different forms, from being a member of a more broadly defined music culture (popular music, Western art music, and so on) to being a highly accomplished performer in a particular repertoire. The aim of the experiment reported in the present paper was to investigate the latter, that is, effects of genre-specific musical expertise on micro-level timing and synchronization. To this end, we asked: Do expert musicians from different musical genres perceive musical sounds differently? And when asked to synchronize with these sounds, do they do so differently? Previous research has focused on general musical expertise or on possible effects of instrument training (e.g., percussionists vs. other musicians). This study aims at providing new insights into possible effects of active long-term musical enculturation and skill acquisition on basic perceptual auditory skills. At a more general level, it sheds light on the important question of how nature and nurture intersect in the development of our perceptual systems.

In order to synchronize with each other, musicians must perceive the temporal location of each other’s sounds—but this perception involves more than detecting the acoustic onset of a sound. The perceived temporal location for a sound is known as its *P-center*, a term derived from studies of perceived onset times of speech sounds in phonetics (Morton, Marcus and Frankish, 1976), and extended to the perception of musical sounds (Gordon, 1987; Villing, 2010; Vos and Rasch 1981). P-centers typically occur between the acoustic onset and first energy peak in the amplitude envelope of a sound, but a constellation of factors, including not only amplitude rise time, but also center frequency, sound duration, and spectral composition/timbre can affect P-center perception. Moreover, P-centers are best regarded not as points in time after the onset of a sound, but rather as a probability distribution of a sound’s perceived temporal location (Danielsen et al., 2019; Wright, 2008). P-centers thus have both a peak and a spread, which Danielsen (2010, 2019) has characterized as the “beat bin” in musical contexts. Thus, to state our first research question more precisely, “do expert musicians from different musical genres perceive the P-center/beat bin of a given sound differently?”

The role of musical training in developing the perception and production abilities needed for fine-grained timing and synchronization of sounds has been investigated in several studies, using both behavioral and neuroscientific methods. Regarding timing production*,* that is, the act of accurately synchronizing a self-produced sound (such as a tap) with an external sound source, research has shown that musicians do this with *lower variability* than non-musicians (Cameron and Grahn, 2014; Danielsen et al., 2019; Fujii et al., 2011; Krause et al., 2010; Manning & Schütz, 2015; Manning, Harris & Schütz, 2017; Manning, Simoniski & Schutz, 2020; Matthews et al., 2016; Repp & Doggett, 2007; Repp 2010; Skansaar et al., 2019). Musicians also show less *asynchrony* when tapping to an isochronous auditory sequence (Cameron & Grahn, 2014; Danielsen et al., 2019; Repp, 2010; Repp & Doggett, 2007). Krause et al. (2010) showed that percussionists showed particularly low asynchrony when tapping to an auditory metronome, and studies by Manning et al. yielded similar results when comparing percussionists with non-musicians (Manning & Schütz, 2015; Manning, Harris & Schütz, 2017). Musicians also tap with less variability to more complex rhythms (Chen et al., 2008). Overall, research into timing production shows that musicians generally have more precise timing and synchronization abilities.

When judging temporal precision without producing a sound, musicians have also been found to outperform non-musicians (Rammsayer & Altenmüller, 2006; Repp, 2010). They are better at judging when two sounds are in perfect synchrony (Danielsen et al., 2019; Matthews et al., 2016) and at discriminating timing deviants in a rhythmic sequence (Jones et al., 1995; Jones & Yee, 1997; Yee et al., 1994). However, in some of the studies by Manning et al. referenced above, there was no difference between musicians and non-musicians in detecting timing deviants when listening without moving (Manning, Harris & Schütz, 2017; Manning, Simoniski & Schutz, 2020). Matthews et al. (2016), however, while finding a clear difference between non-musicians and musicians, found no differences between drummers, singers, pianists and string players in a beat alignment perception task. Similarly, van Vugt & Tillmann (2014) found lower thresholds for detecting a timing delay for musicians than non-musicians, but no difference between the two musician groups (pianists and brass players). Importantly, none of the above studies were controlled for differences in the musical genres in which the musicians performed and/or the non-musicians preferred, which means that genre-specific musical training could be a confound in these studies, e.g., jazz versus classical pianists.

Results from neuroscience support the behavioral findings. The primary mechanisms thought to underlie musical expertise is a strong link between sound perception and movement production, or auditory–motor integration (Zatorre et al., 2007). Accordingly, when performing timing tasks, musicians show greater engagement of the cerebellum compared to non-musicians (Chen et al., 2008; Grahn & Brett, 2007). Musicians also show larger mismatch negativity (MMN) in response to timing deviants (Rüsseler et al., 2001) and have been found to allocate more attention to timing tasks than non-musicians as indexed by pupillometry (Skansaar et al., 2019). Generally, there is solid evidence for changes in both structure and function in musically trained individuals compared to untrained persons (Leipold, Klein & Jäncke, 2021), which probably reflect adaptations or plasticity related to increased abilities for musical perception and performance (for review, see Herholz & Zatorre, 2012).

Although musicians have superior timing abilities, this does not mean that timing asynchronies or variability in timing are to be avoided in all musical situations. Interview studies with musicians from various genres show that optimal timing can vary considerably from genre to genre. In EDM (Brøvig-Hanssen et al., 2021; Butler, 2006), disco (Danielsen, 2012), and jazzfunk (Câmara, 2016), very tight synchronisation among all instruments is the overall ideal. In jazz (Butterfield, 2010; Monson, 1996) and funk (Danielsen, 2006), by contrast, one often finds timing asynchronies between bass and kick drum around 20-30 milliseconds or more. Furthermore, in many styles of hip-hop, neo-soul and contemporary R&B overall there is a preference for rather loose timing, as there one may find timing asynchronies of up to 80-90 milliseconds. This is clearly audible and close to a 32^nd^ note’s duration at a tempo of 90 beats per minute, which is a tempo typical of these styles (Bjerke, 2010; Carlsen & Witek, 2010; Danielsen 2010, 2019). Scandinavian traditional fiddle music in the so-called *springar* tradition is an even more extreme example, as it allows for, and even prefers, extremely flexible timing; in *springar* beat durations may vary with up to 200 milliseconds within one bar (Bengtsson, 1974; Blom, 1981; Groven, 1971; Johansson 2010a, 2010b, 2017a, 2017b; Kvifte, 2005, 2007). Thus being trained in a certain genre thus represents a very specialised form of timing experience and a particular listening biography, and both have the potential to shape music-perceptual abilities.

Experimental investigations of the effects of genre-specific musical training are extremely scarce but the results point to familiarity with the music being key to perception. Senn et al. (2018) did a groove rating study with 248 reconstructed drum patterns from different popular music styles (pop, rock, funk, heavy metal, rock'n'roll, hip hop, soul, R&B) with 665 participants. They found that the listeners' taste, musical biographies and expertise had a strong effect on their groove ratings which far exceeded other factors such as syncopation and beat salience. Vuust and colleagues (2012) tested musicians from three different genres (classical, jazz, rock/pop) and non-musicians by recording the pre-attentive MMN response to changes in six different musical micro-features (pitch, timbre, location, intensity, pitch slide, and timing). They observed a more frontal MMN to pitch and location compared to the other deviants in jazz musicians and left lateralization of the MMN to timbre in classical musicians, which they interpreted as support for musicians’ brain being shaped by the type of training, musical style/genre, and listening experiences. A recent study by Kliuchko, et al. (2019), employing the same paradigm, found generally larger MMN amplitudes in response to deviants typical of jazz in jazz musicians, but not in classical musicians, as compared to non-musicians and amateurs, suggesting that long-lasting, active experience of a musical style is associated with neural priors for the sound features of the preferred style, in contrast to passive listening. Previous studies have also shown that musicians exhibit particular sensitivities to timbres with which they have special long-term auditory experience (Fujioka et al., 2006; Pantev et al., 2001a). Margulis et al., (2009) examined this by fMRI and found that an extensive cerebral network was activated when violinists listened to violin music and flutists to flute music, compared to when they listened to music by the other instrument. The network implicates increased sensitivity to musical syntax (activation in BA 44), timbre (auditory association cortex), and sound–motor interactions (precentral gyrus) for own music and suggest that the musicians’ specialized training particular “listening biography” shape musical perception in several important ways.

There is also a cluster of studies that have investigated the effects of preference and familiarity on musical recognition and classification, showing that musical genre and expressive character can be identified after only 250-500 ms (Filipic et al., 2010; Gjerdingen & Perrott, 2008; Krumhansl 2010; Schellenberg et al., 1999). In an ERP study, Istok et al. (2013) investigated successive evaluative processing stages in fans of Latin-American music and heavy metal in response to music from both genres and found similar patterns for the preferred genre compared to the non-preferred genre in both groups. They suggest that the affective valence of a piece of music may spontaneously modulate early processes of music categorization even when no overt preference judgement is required.

One’s musical and broader cultural background can also influence the perception of auditory stimuli at a basic level. Deutsch (1991, 1997) famously reported on how a speaker’s native language/dialect can bias the perception of pitch height in ambiguous stimuli (the “Tritone Paradox”), and a listener’s native language can influence the grouping of nonverbal rhythms (Iversen et al. 2008; Kusumoto & Moreton, 1997; Patel et al., 2006). Likewise, tonal language speakers have been found to outperform non-tonal language speakers in pitch discrimination tasks (Hu et al., 2020). In a similar way, expert knowledge of a particular form of music might work as a native musical “language” that profoundly affects the perception and cognition of other less familiar musical “languages”. Hannon, Soley & Ullal (2012) found that Turkish listeners were better than Americans at detecting deviants in complex rhythms common in Turkish music, whereas there were no differences between groups for complex rhythms that were non-familiar to both groups. Precision in meter processing has also been found to depend to a great extent on the amount of experience with specific meters (Ullal-Gupta, Hannon & Snyder, 2011). Furthermore, a cross-cultural tapping study by Polak et al. (2018) demonstrated that expert Malian musicians were able to precisely synchronize with and maintain a more complex, but culturally-specific rhythmic prototype (i.e., a rhythm with a ratio of 58:42), while the performance of musicians from Germany and Bulgaria devolved toward a simpler 2:1 ratio typical of rhythms in Western and other musical traditions. Similar results have been found in a larger study by Jacoby et al. (2021) that involved participants from 15 countries on five continents, spanning modern societies and traditional indigenous populations belonging to 39 subgroups with varied musical expertise.

The different strands of research reported above all point to the significant role of enculturation in shaping our perception and production of musical rhythm. That is, not only do expectations derived from our immediate context produce a bias regarding how to interpret sensory information, the neural plasticity of our brains allows enculturation to shape our perceptual information in a more fundamental way by modulating our basic, bottom-up perceptual mechanisms via top-down processes (Engel, Fries & Singer, 2001; Gilbert & Sigman, 2007).

In the present study we probe the effects of active, long-term engagement with specific musical genres on musicians’ fine-grained rhythmic timing and synchronization skills in terms of their perceptions of the location and variability of the P-centers in a variety of musical and non-musical sounds. To this end, we recruited three groups of active musicians/producers with high levels of musical expertise within three musical genres where rhythm is at the core: jazz, Scandinavian traditional fiddle music (in the following named “folk”), and computer-based popular music styles, such as, electronic dance music, electro-pop, trap and hip-hop (in the following named “producers”). We asked them to both synchronize a click track as well as tap along with stimuli made of sounds from their own genre, the two other musical genres, and a set of genre-neutral quasi-musical sounds. Each stimulus category had a balanced 2 X 2 design of the acoustic factors of Attack/Rise Time (slow vs. fast) and Duration (short vs. long). The primary aim was to probe effects of genre-specific musical training and concomitant enculturation. Our research hypotheses were as follows:

(1) As to the *genre-specific sounds*, we hypothesized that participants would be more consistent, that is, show less variability, when aligning clicks (perception task) and when synchronizing taps (production task) to sounds that are characteristic of the genre in which they are trained, compared to sounds from the other musical genres.

(2) We expected an effect of genre expertise on the different participant groups’ approach to timing in general, measured as click and tap variability for the *genre-neutral sounds*. In particular, we expected to see higher variability among the folk musicians compared to the two other groups.

(3) Finally, in line with the results of our previous studies (Danielsen et al., 2019; London et al., 2019) we expected that longer rise time and longer duration would generally lead to later P-center (Morton, Marcus & Frankish, 1976) and a wider beat bin (Danielsen, 2010), that is, a later synchronization point and more flexibility regarding “correct” synchronization, evidenced by greater variability in click and tap timing.

**Methods**

#### **Participants**

60 professional musicians and producers were recruited from the Oslo area. 20 had a background in jazz (jazz, median age = 35 years; 25% female), 19 in Scandinavian fiddle music, (folk, median age = 36 years; 42% female), and 21 were producers working with computer-based dance music genres. One participant in the producer group was excluded due to erratic tapping, reducing the N in this group to 20 (producers, median age = 26 years; 20% female). A power calculation conducted in G*Power (Faul et al., 2007) indicates that for the planned repeated measures ANOVAs, 16 participants would be needed to have 80% power for detecting a medium sized effect when employing the traditional .05 criterion of statistical significance, whereas 54 participants are needed to detect any between-groups effects. (Expected effect sizes are based on previous research, see Danielsen et al., 2019.) After the tests, participants completed a questionnaire regarding their musical training and listening practices. Results are summarized in Table A1 in the Appendix. Written informed consent was obtained and each participant received a gift card (value 200 NOK) for their participation in the experiment.

#### **Stimuli**

The stimuli consisted of three stimulus categories, each of which had a 2x2 design of the two acoustical factors: Attack (fast/impulsive vs. slow/gradual rise time) and Duration (of the stimulus sound, as opposed to the stimulus IOI). Two categories were musical sounds commonly used in the participants’ genres. The first (Organic) consisted of a kick drum (fast-short) and an electric bass sound (fast-long) typically used in jazz, and two fiddle sounds (slow-short and slow-long) typical of Scandinavian fiddle music. The second category consisted of synthesized sounds (Electronic) typically used in EDM and hip-hop: an 808 kick drum (fast-short) and three different synth bass sounds (fast-long, slow-short, slow-long). Manual and computational measurements^[[1]](#footnote-1)^ of the waveforms are reported in Table A2 in the Appendix. The third category (Neutral) consisted of four quasi-musical sounds that represent a fully controlled, balanced design of Attack (fast=3 ms, slow=50 ms) and Duration (short=100 ms, long=400 ms), see Table A2 in the Appendix. The sounds were generated in Max 7, using white noise and bandpass-filters with a Q-factor of 10. The amplitude of the sound files was scaled linearly from 0 (beginning of file) to 1 (at the indicated rise time), immediately followed by a linear decay to silence at the end of each sound file. A click sound (i.e., the same as the click probe in the click-alignment task) was also included amongst the stimuli. Because there is no way of arriving at an objectively equal level of loudness for sounds with these different sonic characteristics, the relative loudness level of the different sounds was adjusted by ear by two of the experimenters.

**Apparatus and procedure**

During the click alignment (“CLICK”) trials the participants aligned a click track with a loop of the target stimulus. Click and the stimulus sound were both looped at 600ms interval (tempo=100 bpm) and the task was to move the click track, that is, the entire click loop, in time so that the click loop and stimulus loop were perfectly aligned. The two loops started with a random offset of +/- 100-200 ms. In each trial, participants manipulated the timing of the click track by moving an on-screen cursor using the mouse and/or arrow keys (one key click = 1 millisecond). Participants were also able to adjust the volume of the click track. When satisfied that the target stimulus was synchronized with the click track, participants moved to the next trial. Following two practice trials, participants heard each target stimulus three times for a total of 39 trials. The order of stimulus presentation was randomized but constrained so that participants never heard the same stimulus on back-to-back trials. There was no time limit for each trial in the click alignment task, and on average the folk musicians used 51 seconds per trial, whereas the jazz musicians and producers used 31 and 29 seconds, respectively. The overall time for each participant to perform all CLICK trials varied from 30 to 60 minutes.

The folk participants completed the CLICK task using HP Compaq Elite 8300 computers (3.2 GHz Intel Core i5-3470, Windows 10 Enterprise), listening via Beyer Dynamic DT 770 Pro headphones at a comfortable intensity that could be further adjusted by the participant. The jazz and producer participants completed the CLICK task using a MacBook Pro computer (3.1 Ghz Intel core i7, OSX 10.11.16), listening via Beyer Dynamic DT 770 Pro at a comfortable intensity that could be further adjusted by the participant. Stimuli were presented using a custom-made patch written in Max 7 (http://www.cycling74.com), which also recorded participants’ responses, in total 2346 click locations (13 stimuli x 3 trials x 60 participants).

Each participant’s responses were averaged across the three trials to produce a p-center for each stimulus per participant; p-centers are reported in milliseconds relative to the physical onset of each stimulus. 18 trials were excluded because the click occurred during silence, that is, at least 50 ms outside of the sound. The standard deviations of the three trials for each stimulus for each participant were also calculated. The grand averages of participants’ p-centers and standard deviations were used as measures of the overall CLICK p-center and variability for each stimulus.

In the Tapping trials (“TAP”), the task was to tap along using a pair of clave sticks in synchrony with the target stimulus, again looped at a 600ms interval. Each loop repeated for 20 seconds. Participants were given two practice trials to gain familiarity with the clave sticks as well as with the task at hand. The presentation of the 13 target stimuli was randomly ordered. Participants took from 5 to 10 minutes to finish the Tapping trials.

In the TAP task participants used acoustically transparent headphones (Koss PortaPro) which allowed them to clearly hear their tapping during those trials. To eliminate timing latencies during the Tapping task, the stimulus was split and routed both to participants’ headphones and to a mono recording channel on an audio interface (RME Babyface Pro or RME Fireface UCX); tapping data were recorded on another mono channel using a Shure SM57 unidirectional microphone. A MATLAB script was used to identify tap onsets, as the time point where the rectified tapping audio waveform first exceeded a predefined threshold close to the noise floor. For each registered tap, the time difference between its detected onset and the first zero crossing of the closest stimulus sound was calculated.

Each tapping trial involved a 20-second presentation of a looped stimulus; data were collected from 20 consecutive taps, beginning with the fifth tap of each trial. Tap/Stimulus onset asynchronies were averaged to calculate the TAP p-center for each stimulus. One series by one participant in the folk group had only 9 taps and revealed erratic tapping and was thus excluded. For this particular series we used the mean of means of all participants in the relevant genre group for this sound in the statistical analysis. Of the remaining 767 series (59 participants x 13 stimulus sounds) three series had fewer than 24 registered taps; in those cases all registered consecutive taps from the fifth tap were used, that is, 10, 18 and 19 taps, respectively. The standard deviation of the tap/stimulus asynchronies in each trial was also calculated. As in the CLICK task, the grand averages of participants’ p-centers and standard deviations were used as measures of the TAP p-center and TAP variability for each stimulus.

The order in which participants completed the two tasks was counterbalanced. Between or after experimental tasks, participants completed the background questionnaire. For the folk CLICK trials, eight to ten participants simultaneously ran trials at individual workstations in the University of South-Eastern Norway’s PC suite. The jazz and producer CLICK trials as well as all TAP trials were conducted as individual sessions in the participant’s home, studio, or a quiet workspace. Participants were encouraged to proceed through the experiment at their own pace and to take breaks as needed. One of experimenters waited nearby should any questions/problems arise.

**Statistical analyses**

We conducted a mixed RM ANOVA with Attack (fast, slow), Duration (short, long), Task (click alignment, tapping), and Stimulus Category (organic, electronic, neutral) as within-subjects and Genre expertise (folk, jazz, producers) as between-subjects independent variable. Follow-up ANOVAS were conducted for each Stimulus category, separately. All tests were conducted with (a) mean click/tap location relative to onset (‘p-center’) or (b) standard deviation of location (‘variability’) as the dependent variables. Multiple comparisons were Bonferroni corrected. Violations of sphericity were corrected using Greenhouse-Geisser. The statistical tests were performed in SPSS (ver. 27) (IBM).

**Results**

**Preliminary Data analysis**

As a preparation for the main ANOVA, we tested whether age should be included as a covariable. The producer group is, on average, younger than the folk musicians, and there is evidence that temporal acuity decreases with age (Walton, 2010). We conducted a Pearson’s correlation test between age and the variability results for the click sound in the CLICK task, but found no significant correlation (r = -.059, N = 60, *p* = .653). As Musical Expertise is related not only to musical genre, but also to one’s particular musical instrument (e.g., one can play electric guitar in a wide range of styles), we also explored the effect of participants’ particular musical instruments via an ANCOVA analysis which included participants’ primary instrument as a co-variate (substantial training on a secondary traditional instrument [more then 10 years and currently active] was included for producers). We categorized instruments in terms of their sound production characteristics, namely “tapper/plucker/”—those characterized by a sharp attack and then steady decay—versus “breather/bower”—instruments (and voice) which have softer attacks and more sustained tone. 58% of the folk musicians are breather/bowers (i.e., fiddle players and singers) compared to 30% in the jazz and producer groups, and the jazz musicians had more tappers/pluckers (70 % were drummers, guitarists, bass players, and keyboard players) compared the producer (50%) and folk (42%) groups (see also Table A1 in the appendix). However, as type of instrument training is not independent of genre, this covariate did not clarify the data analysis. Thus, we restrict our between-groups analysis to the three categories of Genre Expertise outlined above (Folk, Jazz, and Producer). Further comments on the effect of instrument are given in the discussion/notes for future research.

Descriptive statistics of the mean location and variability (per stimulus) for both click alignment and tapping trials are given in Table A3 in the Appendix.

**Main Effects of Task and Acoustic Factors**

We conducted a (2x2x2x3)x3 mixed ANOVA with Task (CLICK vs. TAP), Attack (fast vs. slow), Duration (short vs. long), and Stimulus Category (organic vs. electronic vs. neutral) as within-subjects variables, and Genre/Group (folk vs. jazz vs. producers) as a between-subjects variable. The two dependent variables are mean p-center location and p-center variability. Table 1 lists the Main Effects (MEs) and all two-way interactions for the ANOVA. Three- and four-way interactions have been omitted, as they were mostly non-significant; the few that were present were due to the effect of the long-slow fiddle sound discussed in detail below.

**Table 1.** *Main and two-way interaction effects of the Mixed ANOVA for P-center location and P-center variability*

|  | P-center | | |  | P-center variability | | | |  | |
| --- | --- | --- | --- | --- | --- | --- | --- | --- | --- | --- |
|  | *F* | *df* | *p* | η_p_^2^ | *F* | *df* | *p* | *η_p_^2^* | |  |
| Task | 22.749 | 1 | **<0.001** | 0.289 | 19.088 | 1 | **<0.001** | 0.254 | |  |
| Task * Group | .651 | 2 | .525 | 0.023 | 2.322 | 2 | 0.107 | 0.077 | |  |
| Error (Task) |  | 56 |  |  |  | 56 |  |  | |  |
| Attack | 583.685 | 1 | **<0.001** | 0.912 | 186.715 | 1 | **<0.001** | 0.769 | |  |
| Attack * Group | 13.487 | 2 | **<0.001** | 0.325 | 2.533 | 2 | 0.087 | 0.084 | |  |
| Error (Attack) |  | 56 |  |  |  | 56 |  |  | |  |
| Duration | 386.544 | 1 | **<0.001** | 0.873 | 78.945 | 1 | **<0.001** | 0.585 | |  |
| Duration * Group | 16.803 | 2 | **<0.001** | 0.375 | 1.272 | 2 | 0.288 | 0.043 | |  |
| Error (Duration) |  | 56 |  |  |  | 56 |  |  | |  |
| StimCat | 192.956 | 1.350 | **<0.001** | 0.775 | 49.460 | 1.643 | **0.001** | 0.469 | |  |
| StimCat * Group | 13.899 | 2.700 | **<0.001** | 0.322 | 3.909 | 3.286 | **0.009** | 0.123 | |  |
| Error (StimCat) |  | 75.596 |  |  |  | 92.019 |  |  | |  |
| Task * Attack | 4.128 | 1 | **0.047** | 0.069 | 58.490 | 1 | **<0.001** | 0.511 | |  |
| Task * Attack * Group | 0.023 | 2 | 0.977 | 0.001 | 1.813 | 2 | 0.173 | 0.061 | |  |
| Error (Task*Attack) |  | 56 |  |  |  | 56 |  |  | |  |
| Task * Duration | 1.518 | 1 | 0.223 | 0.026 | 13.740 | 1 | **<0.001** | 0.197 | |  |
| Task * Duration * Group | 0.306 | 2 | 0.738 | 0.011 | 0.434 | 2 | 0.650 | 0.015 | |  |
| Error (Task*Duration) |  | 56 |  |  |  | 56 |  |  | |  |
| Attack * Duration | 170.882 | 1 | **<0.001** | 0.753 | 24.782 | 1 | **<0.001** | 0.307 | |  |
| Attack * Duration * Group | 11.492 | 2 | **<0.001** | 0.291 | 0.821 | 2 | 0.445 | 0.028 | |  |
| Error (Attack*Duration) |  | 56 |  |  |  | 56 |  |  | |  |
| Task * StimCat | 6.394 | 1.520 | **0.002** | 0.102 | 5.567 | 1.494 | **0.010** | 0.090 | |  |
| Task * StimCat * Group | 0.385 | 3.040 | 0.766 | 0.014 | .639 | 2.989 | 0.591 | 0.022 | |  |
| Error (Task*StimCat) |  | 85.118 |  |  |  | 83.683 |  |  | |  |
| Attack * StimCat | 120.644 | 1.363 | **<0.001** | 0.683 | 42.532 | 1.544 | **<0.001** | 0.432 | |  |
| Attack * StimCat * Group | 12.347 | 2.726 | **<0.001** | 0.306 | 3.345 | 3.108 | **0.021** | 0.107 | |  |
| Error (Attack*StimCat) |  | 76.314 |  |  |  | 87.029 |  |  | |  |
| Duration * StimCat | 139.844 | 1.207 | **<0.001** | 0.714 | 33.533 | 1.413 | **<0.001** | 0.375 | |  |
| Duration * StimCat * Group | 11.213 | 2.415 | **<0.001** | .286 | 3.257 | 2.827 | **0.028** | 0.104 | |  |
| Error (Duration*StimCat) |  | 67.608 |  |  |  | 79.145 |  |  | |  |

*All results involving StimCat (Stimulus Category) corrected using Greenhouse-Geisser estimates of sphericity. Significant results in bold.*

As can be seen in Table 1, there are significant main effects for the acoustic factors of Attack and Duration, as well as Task (CLICK vs. TAP) for both P-center location and variability, replicating our previous studies (Danielsen et al., 2019; London et al., 2019). As can be seen in Figure 1, slow attacks led to later P-centers and greater variability, as did longer durations. The effect of Task was most pronounced in terms of variability, with greater effects of stimulus category in the CLICK task rather than in the TAP task (see further comments in the discussion).


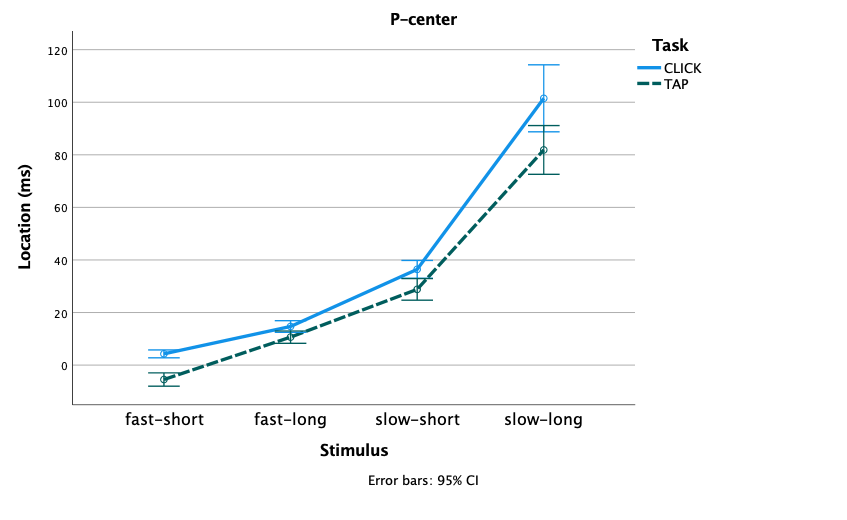

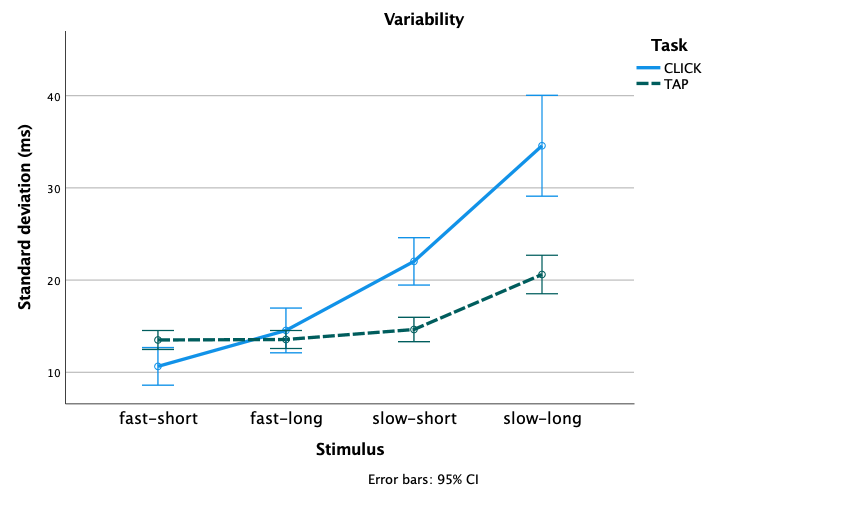


**Figure 1.** Plots of mean p-center location relative to stimulus onset (left) and variability (right) for all combinations of the factors Attack (fast, slow) and Duration (short, long), separated by task. Main effects of Attack and Duration as well as their interaction were significant (p < .001) across tasks for both P-center and variability. Error bars: 95% CI.

**Effects of Genre Expertise and Stimulus Category**

Genre expertise showed a main effect on both p-center, (*F*(2,56) = 9.626, *p* < .001; η_p_^2^ = .256), and variability (*F*(2,56) = 7.964, *p* = .001; η_p_^2^ = .221). Average p-center locations were 26 ms after stimulus onset for Producers, 37ms for Jazz musicians, and 40ms for Folk musicians. Pairwise comparisons were significant for Producers and Jazz musicians (*p = .*005) and Producers and Folk musicians (*p* = .001); the difference between folk and jazz musicians was not significant (Bonferroni correction applied). Average p-center variabilities were 15ms for the producers, 18ms for the jazz musicians, and 22ms for the folk musicians. The difference in variability between the Producers and Folk musicians was significant (*p* = .001; Bonferroni correction applied); no other differences were significant. There were significant interactions between Genre expertise and the acoustic factors of attack and duration for p-center location, but not for p-center variability (see Table 1). There were no significant interactions between of Genre expertise and Task.

There was a significant main effect of Stimulus Category (organic, electronic, neutral) on p-center location and variability (see Table 1). Post-hoc pairwise comparisons show that the p-center for the organic sounds is on average 19 ms later than the electronic sounds, and 46 ms later than the neutral noise sounds, respectively (all significant at *p* < .001). The difference between electronic and neutral sounds’ p-center (27 ms) was also significant (*p* < .001). The variability was also 7 ms higher for the organic sounds compared to both electronic and neutral sounds (both significant at *p* < .001). The difference in variability between electronic and neutral was not significant (*p* = .825).

There was significant interaction between Stimulus Category and Genre Expertise for p-center location (see Table 1). As can be seen in Figure 2, the main effect is driven by the differences in synchronization with respect to the organic sounds: in both CLICK and TAP tasks, mean p-center location was latest for the folk musicians, earliest for the producers, and with the jazz musicians in the middle.


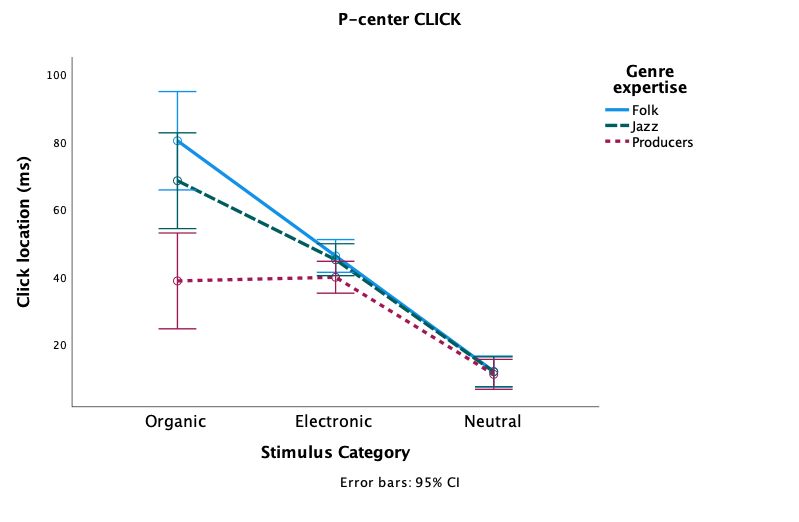

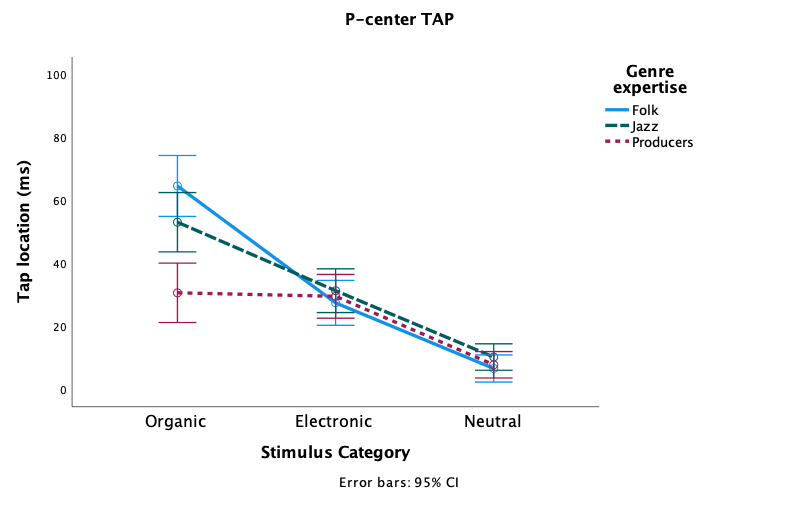


**Figure 2*.*** Plots of mean p-center location relative to stimulus onset by Stimulus Category for CLICK (left) and TAP (right) tasks, separated by participant genres. Error bars: 95% CI.

There was also a significant interaction between Stimulus Category and Genre Expertise for variability (see Table 1). Figure 3 shows that, again, the effect is mainly caused by increased differences between groups in response to the organic sounds.


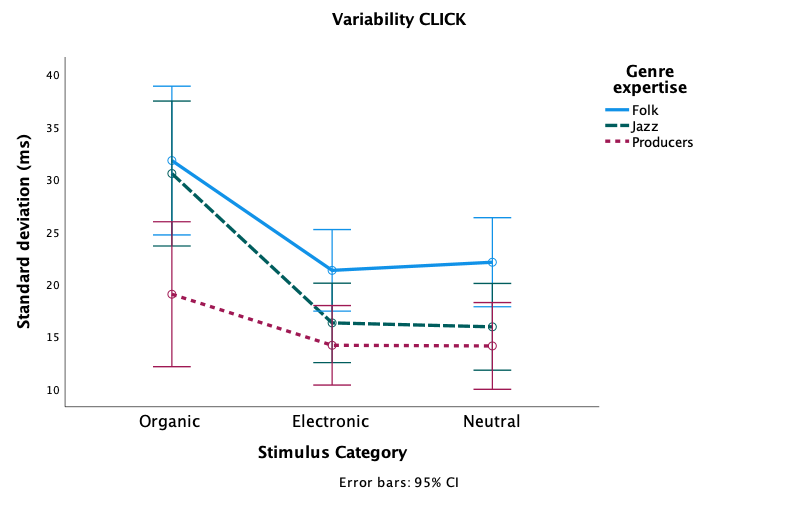

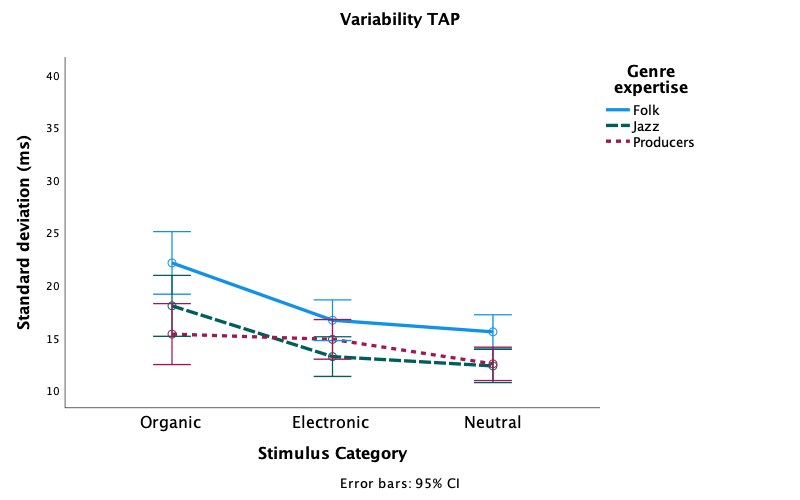


**Figure 3.** Plots of mean p-center variability by Stimulus Category for CLICK (left) and TAP (right) tasks, separated by participant genres. Error bars: 95% CI.

**Effects of Genre Expertise within Specific Stimulus Categories**

To further examine the effect of individual stimuli within each stimulus category, a series of (2x4)x3 Mixed ANOVAs was run for each stimulus category (Task = CLICK vs. TAP; Stimulus = 4 levels; Group/Genre Expertise = Folk, Jazz, Producer); main effects and two-way interactions are given in Table 2 (three-way interactions were not reported, as none were significant).

**Table 2.** Results of Mixed ANOVAs for individual stimulus categories.

|  | P-center | | | | P-center variability | | |  | |  | |  |
| --- | --- | --- | --- | --- | --- | --- | --- | --- | --- | --- | --- | --- |
|  | *F* | *df* | *p* | η_p_^2^ | | *F* | *df* | | *p* | | *η_p_^2^* | |
| **Organic Across Tasks** |  |  |  |  | |  |  | |  | |  | |
| Task | 10.566 | 1 | 0.002 | 0.159 | | 14.407 | 1 | | <0.001 | | 0.205 | |
| Task * Group | 0.385 | 2 | 0.682 | 0.014 | | 1.333 | 2 | | 0.272 | | 0.045 | |
| Error(Task) |  | 56 |  |  | |  | 56 | |  | |  | |
| Stimuli | 174.877 | 1.222 | <0.001 | 0.757 | | 69.000 | 1.412 | | <0.001 | | 0.552 | |
| Stimuli * Group | 14.475 | 2.445 | <0.001 | 0.341 | | 3.116 | 2.824 | | 0.033 | | 0.100 | |
| Error(Stimuli) |  | 68.454 |  |  | |  | 79.060 | |  | |  | |
| Task * Stimuli | 5.254 | 1.180 | 0.020 | 0.086 | | 8.137 | 1.312 | | 0.003 | | 0.127 | |
| Error(Task*Stimuli) |  | 66.063 |  |  | |  | 73.469 | |  | |  | |
| **Organic CLICK** |  |  |  |  | |  |  | |  | |  | |
| Stimuli | 97.933 | 1.140 | <0.001 | 0.636 | | 33.120 | 1.348 | | <0.001 | | 0.372 | |
| Stimuli * Group | 7.329 | 2.280 | 0.001 | 0.207 | | 1.496 | 2.695 | | 0.225 | | 0.051 | |
| Error(Stimuli) |  | 63.851 |  |  | |  | 75.466 | |  | |  | |
| **Organic TAP** |  |  |  |  | |  |  | |  | |  | |
| Stimuli | 169.417 | 1.405 | <0.001 | 0.752 | | 41.378 | 1.376 | | <0.001 | | 0.425 | |
| Stimuli * Group | 15.939 | 2.810 | <0.001 | 0.363 | | 2.631 | 2.752 | | 0.061 | | 0.086 | |
| Error(Stimuli) |  | 78.678 |  |  | |  | 77.060 | |  | |  | |
| **Electronic Across Tasks** |  |  |  |  | |  |  | |  | |  | |
| Task | 38.724 | 1 | <0.001 | 0.409 | | 5.394 | 1 | | 0.024 | | 0.088 | |
| Task * Group | 1.122 | 2 | 0.333 | 0.039 | | 2.450 | 2 | | 0..096 | | 0.080 | |
| Error(Task) |  | 56 |  |  | |  | 56 | |  | |  | |
| Stimuli | 671.196 | 1.608 | <0.001 | 0.923 | | 33.197 | 2.474 | | <0.001 | | 0.372 | |
| Stimuli * Group | 2.295 | 3.216 | 0.079 | 0.076 | | 1.738 | 4.949 | | 0.131 | | 0.058 | |
| Error(Stimuli) |  | 90.061 |  |  | |  | 138.565 | |  | |  | |
| Task * Stimuli | 3.838 | 1.906 | 0.026 | 0.064 | | 22.401 | 2.400 | | <0.001 | | 0.286 | |
| Error(Task*Stimuli) |  | 106.735 |  |  | |  | 134.415 | |  | |  | |
| **Electronic CLICK** | |  |  |  | |  |  | |  | |  | |
| Stimuli | 503.871 | 1.774 | <0.001 | 0.900 | | 32.544 | 2.270 | | <0.001 | | 0.368 | |
| Stimuli * Group | 3.098 | 3.547 | 0.023 | 0.100 | | 1.308 | 4.540 | | 0.267 | | 0.045 | |
| Error(Stimuli) |  | 99.329 |  |  | |  | 127.128 | |  | |  | |
| **Electronic TAP** |  |  |  |  | |  |  | |  | |  | |
| Stimuli | 341.823 | 1.573 | <0.001 | 0.859 | | 32.554 | 2.270 | | <0.001 | | 0.368 | |
| Stimuli * Group | 0.598 | 3.146 | 0.626 | 0.021 | | 1.308 | 4.540 | | 0.267 | | 0.045 | |
| Error(Stimuli) |  | 88.093 |  |  | |  | 127.128 | |  | |  | |
| **Neutral Across Tasks** |  |  |  |  | |  |  | |  | |  | |
| Task | 3.267 | 1 | 0.076 | 0.055 | | 9.997 | 1 | | 0.003 | | 0.151 | |
| Task * Group | 0.334 | 2 | 0.718 | 0.012 | | 1.342 | 2 | | 0.270 | | 0.046 | |
| Error(Task) |  | 56 |  |  | |  | 56 | |  | |  | |
| Stimuli | 122.701 | 2.323 | <0.001 | 0.687 | | 6.319 | 2.628 | | 0.001 | | 0.101 | |
| Stimuli * Group | 3.431 | 4.646 | 0.007 | 0.109 | | 0.728 | 5.256 | | 0.610 | | 0.025 | |
| Error(Stimuli) |  | 130.090 |  |  | |  | 147.163 | |  | |  | |
| Task * Stimuli | 7.582 | 2.538 | <0.001 | 0.119 | | 5.494 | 2.502 | | 0.003 | | 0.089 | |
| Error(Task*Stimuli) |  | 142.116 |  |  | |  | 140.107 | |  | |  | |
| **Neutral CLICK** |  |  |  |  | |  |  | |  | |  | |
| Stimuli | 37.282 | 2.213 | <0.001 | 0.400 | | 6.673 | 2.481 | | 0.001 | | 0.106 | |
| Stimuli * Group | 1.198 | 4.427 | 0.315 | 0.041 | | 0.497 | 4.963 | | 0.777 | | 0.017 | |
| Error(Stimuli) |  | 123.942 |  |  | |  | 138.951 | |  | |  | |
| **Neutral TAP** |  |  |  |  | |  |  | |  | |  | |
| Stimuli | 149.578 | 2.664 | <0.001 | 0.728 | | 0.993 | 1.669 | | 0.362 | | 0.017 | |
| Stimuli * Group | 7.752 | 5.328 | <0.001 | 0.217 | | 1.966 | 3.337 | | 0.118 | | 0.066 | |
| Error(Stimuli) |  | 149.171 |  |  | |  | 93.443 | |  | |  | |

*Task = CLICK vs. TAP; Stimuli = Individual stimuli within each stimulus category (4); Group = Folk vs. Jazz vs. Producer. All results involving Stimuli variables corrected using Greenhouse-Geisser estimates of sphericity.*

Within the Organic Stimuli a main effect was found for Genre Expertise on p-center, (*F*(2,56) = 15.113, *p* < .001; η_p_^2^ = .351). Pairwise comparisons (Bonferroni correction applied) revealed that these group differences were significant (see also figure 2 above): mean click/tap location is on average 38 ms later for folk musicians (*p* < .001) and 26 ms later for jazz musicians (*p =* .001) in comparison to the producers. The difference between folk and jazz musicians was not significant. There was also a significant effect of Stimulus on P-center (see Table 2). All pairwise comparisons (Bonferroni correction applied) were significant (*p* < .001), with one exception, that is, the pair fast-long electric bass–slow-short fiddle (*p* =1.000). There was also a significant interaction between Stimulus and Genre expertise (see Table 2). A closer inspection of the results shows that the long fiddle sound by and large drives the significant differences in p-center between groups, and this is evident in both the CLICK and TAP tasks (see figure 4).


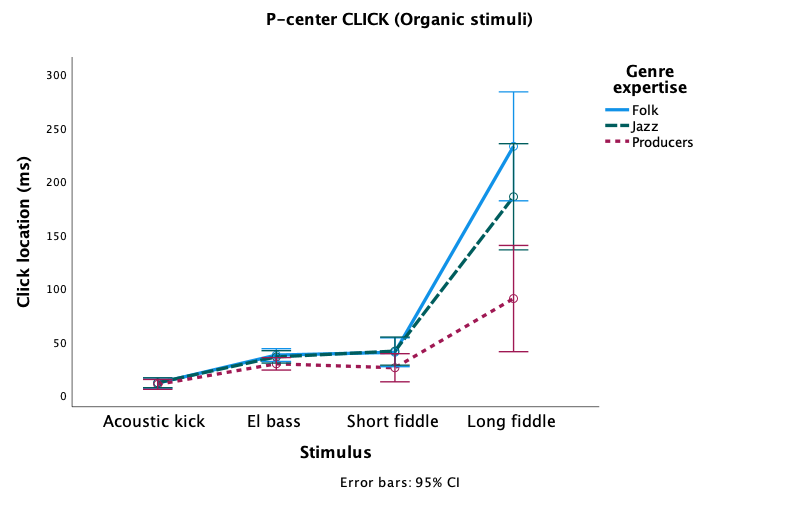

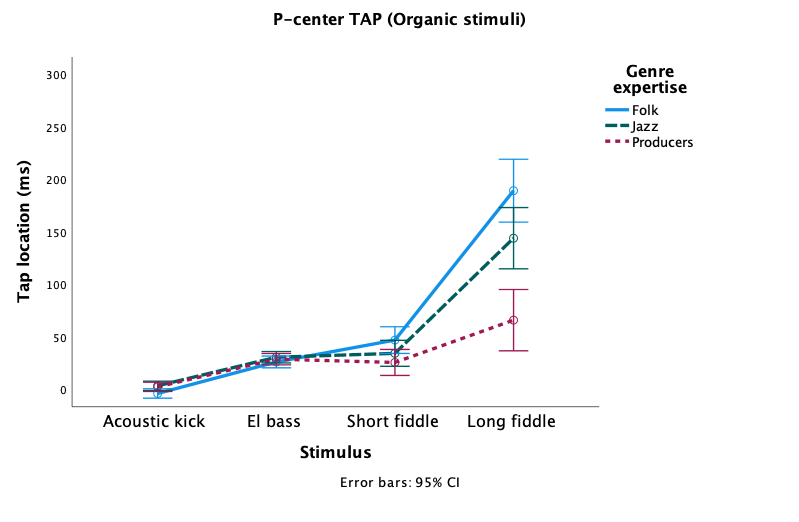


**Figure 4.** Plots of mean p-center locations relative to stimulus onset for individual organic stimuli. CLICK (left) and TAP (right) tasks, separated by participant genres. Error bars: 95% CI.

Regarding variability, the follow-up ANOVA of the Organic stimuli reveals a main effect of Genre expertise (*F*(2,56) = 7.833, *p* = .001; η_p_^2^ = .219). Pairwise comparisons (Bonferroni correction applied) show that the variability is on average 10 ms higher for folk musicians (*p =* .001) and 7 ms higher for jazz musicians (*p =* .020) in comparison to the producers. The difference between folk and jazz musicians is not statistically significant. There is also a significant effect of Stimulus on variability (see Table 2). All pairwise comparisons (Bonferroni correction applied) are significant (acoustic kick–electric bass: *p* = .011, electric-bass–short fiddle: *p* = .002, all remaining comparisons: *p* < .001), and there is significant interaction between Stimulus and Genre expertise, (see Table 2). As Figure 5 indicates, the long fiddle sound (again) is what drives the significant differences in variability between groups, and this is most evident in the CLICK task.


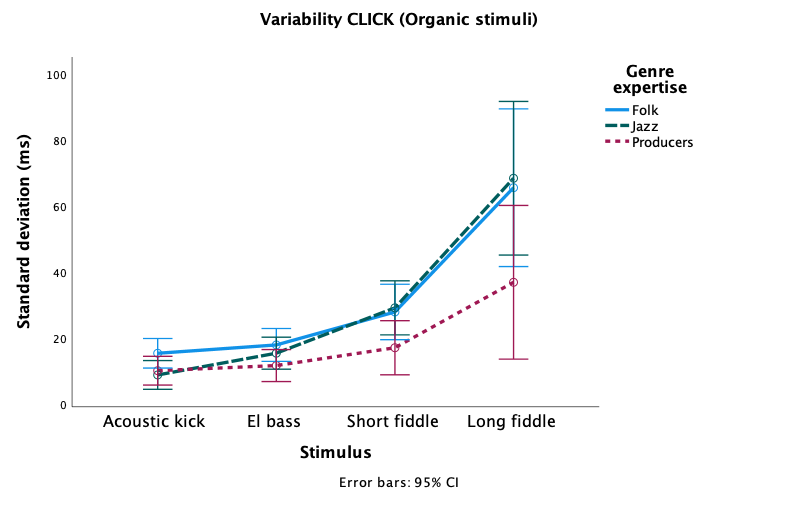

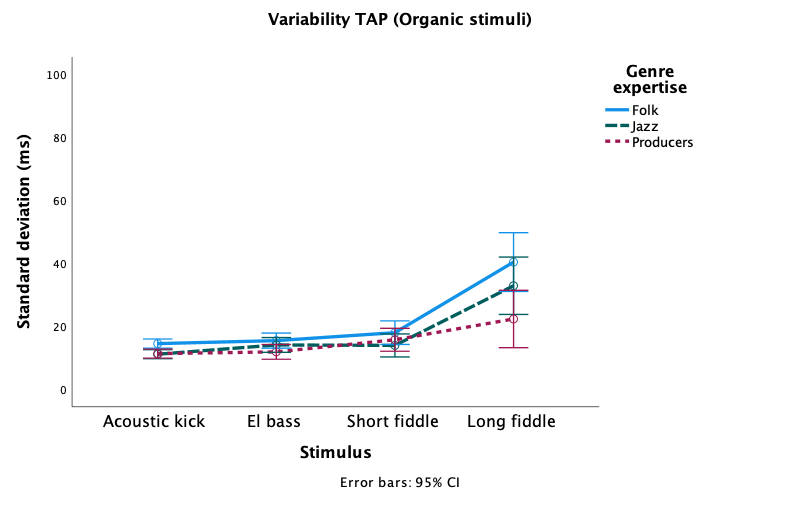


**Figure 5.** Plots of mean variability for individual organic stimuli. CLICK (left) and TAP (right) tasks, separated by participant genres. Error bars: 95% CI.

Figure 6 illustrates the mean P-center locations for each of the three participant groups in relation to the waveform of the long fiddle sound. Figure 7 gives Histograms with the distribution of all click trials with the long fiddle sound for each of the three genre groups, giving a more fine-grained picture of their responses. It shows that a tri-modal distribution of p-center locations is at least latently present in all three groups. The locations of each modal peak (around 100, 200, and 400ms, respectively) correspond to clear inflection points in the amplitude envelope of the sound (see again Figure 6). A calculation of the Shannon entropy of each group’s distribution of clicks reveals that they differ in terms of the flatness/uniformity, with the folk musicians having the flattest distribution and the jazz musicians and producers having more pronounced peaks within their distributions: Folk = -3.1230; Jazz = -2.925; Producers = -2.564.


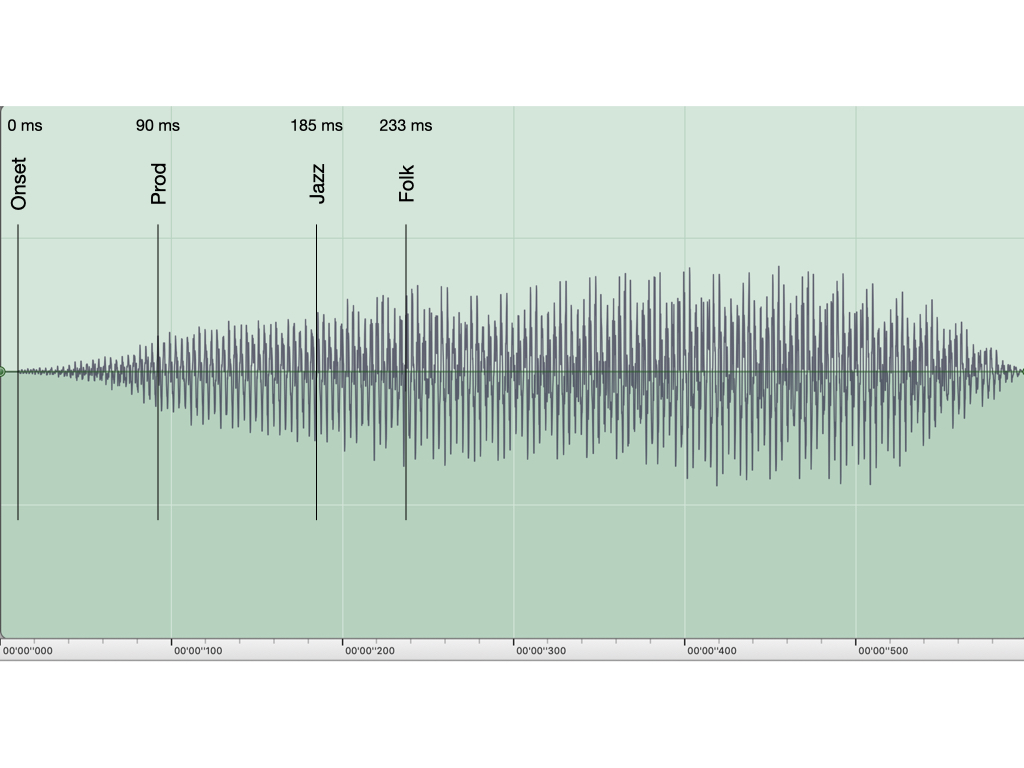


**Figure 6.** Waveform of the long fiddle sound with mean P-center locations in the CLICK task for each of the three participant groups.


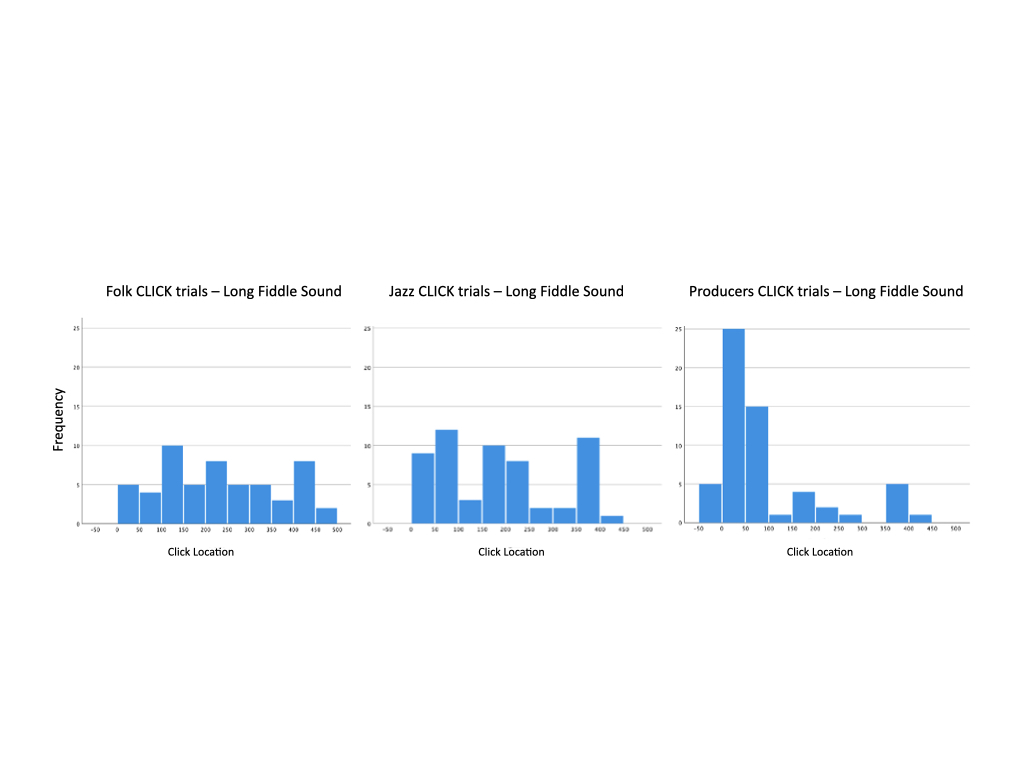


**Figure 7.** Histograms of the distribution of all responses in the CLICK task for the long fiddle sound for each group. Locations are given in milliseconds relative to stimulus onset, and each bin = 50 milliseconds.

The follow-up ANOVAs for the electronic and neutral sounds show no effect of Genre Expertise on p-center, but a main effect of Genre Expertise on variability (Electronic: *F*(2,56) = 4.142, *p* = .021; η_p_^2^ = .129, Neutral: (*F*(2,56) = 6.451, *p* = .003; η_p_^2^ = .187). Pairwise comparisons (Bonferroni correction applied) show significantly higher variability for the folk musicians compared to the producers for both the Electronic (4ms, *p* = .038) and for the Neutral sounds (5ms, *p =* .004). In comparison to the jazz musicians, the folk musicians’ variability was nearly significant for the Electronic sounds (4ms, *p =* .055), and significant for the Neutral sounds (5ms, *p* = .018). Differences in variability between the jazz musicians and producers were not significant.

There is also a significant effect of Stimulus on p-center for both Electronic and Neutral sounds, and a significant effect on variability for both Electronic and Neutral sounds; in addition, a significant interaction was found between Genre expertise and Stimulus category for p-centers of the Neutral sounds (see Table 2). All pairwise comparisons (Bonferroni correction applied) for the p-centers and variability of the Electronic sounds are significant at *p* < .001, except for two variability comparisons (808 kick – fast-short synth bass, *p* = .063, and slow-short – slow-long synth bass, *p* = .020). All pairwise comparisons of p-center for Neutral sounds are significant (fast-long–slow-short at *p* = .007, all remaining comparisons: *p* < .001). Pairwise comparisons for the variability of sounds are significant for fast-short and slow-short (*p* = .002), fast-short and slow-long (*p* = 020), and fast-long and slow-short (*p* = .035); all others are non-significant.

**Discussion**

The aim of the study was to investigate whether training in a particular musical genre influences P-center (measured by average click/tap location relative to sound onset) and beat bin width (as indexed by click/tap variability). To our knowledge no study has previously tested this, even though ethnographic research has shown that optimal timing varies considerably between musical styles (Monson, 1996; Polak et al., 2018; Brøvig-Hanssen et al., 2022). Musicians’ genre expertise thus represents a very specialised form of training and listening history, which might shape their music-perceptual abilities and biases. Accordingly, we expected to see significant differences between the genre groups in our experiment. Our main findings across all sound Stimuli confirmed this hypothesis. We found that producers located the click or tap, on average, earlier than both the folk and jazz musicians, and, as expected based on their training, folk musicians had overall significantly wider beat bins (higher variability) than the producers. There was also a trend (p<.070) for the difference between folk and jazz musicians. It should be noted here that variability differences are generally smaller when reporting results across tasks since, as in our previous studies, the TAP task is less sensitive than the CLICK task to stimulus-driven variability. The difference in how variability data was obtained in the two tasks may explain why. In the TAP task participants performed only one trial for each stimulus (i.e., a looped presentation of a particular sound), while in the CLICK task there were three trials per stimulus. Moreover, the repetitive motion involved in producing the taps with the clave sticks is a "ballistic constraint" on the variability of the inter-tap interval within each series (see also discussion in Danielsen et al. 2019 and London et al. 2019). Accordingly, we see bigger differences in variability between groups in the CLICK task (cf. Figures 3 and 5 above).

As the genre-neutral sounds do not trigger musical-stylistic preferences, we can assume that they best reflect differences in musicians’ bottom-up perceptual timing abilities. For these sounds we found that all groups yield almost identical P-center locations in both tasks (CLICK and TAP) when controlling for Instrument class. However, the folk musicians’ mean variability was significantly higher than the producers across tasks, which supports the hypothesis that difference in variability is associated with the folk musicians’ experiences in performing the temporally-flexible Scandinavian fiddle music versus the producer’s experiences in making groove-based music by way of a digital audio workstation.

Looking into the results for the organic sounds shows that between-group differences are even more apparent when presented with stimuli that engage specialized, group-specific training. The organic sounds consist of “native” sounds for the jazz and folk musicians, that is, acoustic kick drum and electric bass (jazz), and two fiddle sounds (folk). Both fiddle sounds are typical of a style of Hardanger fiddle playing, a musical genre characterized by extreme flexibility as to where two sounds can be synchronized and still be perceived as “in time.” The long fiddle sound produced extreme differences as regards both P-center and variability among the three groups, with extremely “late” P-centers and wider beat bins for the folk and jazz musicians in comparison to the producers. There are a number of possible explanations for this. First are the differences in familiarity with the long fiddle sound and the timing tradition(s) associated with it. Identifying a sound’s genre association happens very fast (Gjerdingen & Perrott, 2008; Krumhansl, 2010; Schellenberg et al., 1999) and might be influencing the bottom-up processing of the P-center. Secondly, the producers working in computer-based genres tend to deal with sounds acousmatically, that is, without paying attention to the sound source, whereas the folk and jazz musicians are most likely responding to both the sound itself and the action(s) of the musician required to produce the sound. The energy peak in the sound occurs when the fiddler has finished the acceleration phase of the bow motion; it is the end of the “attack gesture” of the note. Thus, the musicians (and especially the folk fiddlers) may hear the P-center as when the note has “arrived” at a constant rather than accelerating bow speed (note the amplitude plateau following the folk musicians P-center location in Figure 6). This suggests that they are, at least in part, listening "through" the sound to the action that produces it, hearing the sounds as cues for the coordination of their actions with the sounds of other musicians. Conversely, for the producers the typical musical timing task is discerning the alignment of one sound to another. Synchronization is not primarily tied to the physical action of performance, but rather associated with the act of “nudging” the sound of the blended articulation to produce a desired sonic effect. By contrast, then, the producers are listening “to” the sound, and place the click or tap on the basis of the characteristics of the sound itself, divorced from the actions that produce it.

This makes current the question regarding to what extent genre expertise is (also) related to familiarity with the genre-typical musical instruments, either through own practice, playing with others, or just listening. As mentioned above, most of the folk musicians in the particular Scandinavian folk music tradition that we targeted are breather/bowers (i.e., fiddle players and singers) and in that tradition even plucked string instruments are usually played without “sharp” attacks. In jazz, the picture is more mixed. Comping musicians tend to be tapper/pluckers (i.e., drummers, guitarists, bass players, and keyboard players), whereas singers and wind instruments (breathers) are more flexible, and they can (and do) produce a wide range of sound shapes. The producers’ practice is very different from the folk and jazz musicians, although many of the producers also have training on traditional instruments. Given the design of the present study, it was not possible to investigate whether the training associated with the particular physical properties of different instrument groups represents a form of expertise that can be separated from genre expertise. This is something that we would like to pursue in future research.

Interesting in this regard is also how the three groups differ in terms of the flatness/uniformity of their click distributions, which point to the different locations and widths of the beat bins employed by the different genre groups: the folk musicians have the flattest distribution while the jazz musicians and producers have more pronounced peaks within their distribution. Nonetheless, a tri-modal distribution is evident in all three (see Figure 7 above). The locations of each modal peak (around 100, 200, and 400ms, respectively) seem to correspond to inflection points in the amplitude envelope of the sound (see Figure 6 above). One might thus say that the long, slow fiddle sound affords these multiple locations, and that the folk and jazz musicians tend to use all of them, albeit to a greater or lesser extent, whereas the producers are attracted to the first location for their sense of the sound’s P-center.

The variability results for the organic sounds are perhaps even more surprising, as the folk and jazz musicians produced a *wider* beat bin (11 and 9 milliseconds, respectively) than the producers in response to the organic sounds, despite the fact that they are presumably *more* familiar with these sounds than the producers. Given that previous studies have shown increased temporal acuity as a result of expertise (musicianship), one would expect the lowest variability within each group for the sounds that are most familiar. Our results demonstrate, at least for our stimuli and our participants, that the opposite is rather the case: Both the folk and jazz musicians show high variability (more than 60 milliseconds standard deviation in the CLICK task) for the slow-long fiddle sound, which indicate that they perceive this sound as having a very wide beat bin.

Again, the performers’ greater variability/wider beat bins may be linked to the way in which they afford synchronization. Having a greater “tolerance” (Johansson, 2010b) for synchronization may engender joint action, as some tolerance is necessary for the process of mutual phase correction that is involved in maintaining tight synchrony, such that overall phase error is lower between two human musicians than between a human musician and a metronome (Himberg, 2014; Repp, 2005; Repp & Su, 2012). However, neither tight synchrony nor stable phase relationships between rhythmic layers is something that musicians would necessarily strive for in Scandinavian fiddle music. Even though it is part of a dance music tradition with a recognizable rhythm and meter (Haugen, 2016), Scandinavian fiddle music is ideally performed with a “breathing” rhythmic feel (Johansson 2010a, 2010b, 2017a). Interestingly, several of the folk musicians commented that they found it particularly difficult to synchronize a click to the slow-long fiddle sound. Moreover, the variability found in response to the long fiddle sound is far beyond what is usually found in groove-based performance in jazz contexts (Butterfield, 2010). It is thus likely that the extraordinary wide beat bins in response to the long fiddle sound is related to the aesthetic ideal of performing with flexible timing, and that familiarity with and training in this tradition of Scandinavian fiddle music plays a role beyond general differences between a performance and a production mode.

Summing up, we found small but significant differences between groups of musicians when they respond to neutral sounds that do not trigger any musical-stylistic knowledge. As to the genre-specific sounds, we found a large, genre-related effect in the context of the long fiddle sound. As musical expertise—both in general and instrument-specific—has repeatedly been shown to increase temporal acuity in both perception and production tasks, we hypothesized that familiarity with a genre and its characteristic sounds would correlate with lower variability in P-center location, but we found the opposite to be the case: the folk musicians exhibited the highest variability/the widest beat bins for the long fiddle sound. We suggest several explanations for this, first, that the performance mode of the folk and jazz musicians make them hear the sounds as affordances for actions in a way that the producers don’t do. Secondly, we propose that the top-down genre expertise of the folk musicians and, albeit to a lesser degree, the jazz musicians, influences the bottom-up, acoustically-driven processing for the P-centers of some sounds (i.e., the long fiddle sound). Both behavioral (Deutsch, 1991; Gjerdingen & Perrott, 2008; Senn et al., 2018) and neuroscientific research (Fujioka et al., 2006; Istok et al., 2013; Margulis et al., 2009; Pantev et al., 2001b; Vuust et al., 2012) has made evident that music perception is active, selective and influenced by training and contextual factors. Identifying the long fiddle sound as typical of the particular performance tradition of Scandinavian fiddle music might thus induce not only a late location, but also a perceptually widened beat bin in the appropriately enculturated participants. The producers, on the other hand, who are the least familiar with this fiddle tradition, perceive this sound in a more neutral way, that is, in accordance with its basic acoustical features (early location and lower variability). To the producers the organic sounds are primarily sounds whereas to the folk and jazz musicians they are also affordances for genre-specific forms of joint action.

In short, the effects of genre expertise are manifest at three levels:

- Training has an effect on what seem to be general low-level perceptions of sounds, as evidenced by the differences in p-center variability/beat bin width for the neutral sounds
- Training has an effect on how sounds are heard/grasped in terms of their affordance(s) for action/synchronization, as evidenced by the p-center results for organic sounds.
- Training has an effect as top-down influence on bottom-up processing in terms of activating genre-specific timing ideals, as evidenced by the p-center and variability results for the long fiddle sound typical of the Scandinavian fiddle music tradition.

As a consequence, familiarity with sounds does not necessarily mean lower variability (higher precision). When the sounds strongly signal a genre affiliation and/or the relevant habitual task requires or affords flexibility, then related timing expectations, stylistic rules, and task-specific habits become relevant and influence both the perception and production of what the “correct” timing should be.

**Limitations and future research**

An obvious limitation is that our strongest results are derived from one stimulus in our stimulus set—the long fiddle sound—and thus area for future research involves investigating additional genre-specific sounds, with a wider range of attack and duration characteristics, to see if similar effects are present. In addition, a key question is whether the perceptual effects of performance experience are also developed through training on specific musical instruments versus mere exposure to a particular music genre, or whether performance expertise in the specific genre is required. Honing & Ladinig (2009) designed an experiment specifically to tackle the effect of exposure to music, focusing on sensitivity to expressive timing in music. They found evidence that this sensitivity is more likely to be grounded in active listening (exposure) rather than by formal musical training (expertise). Also, the genre identification studies (Filipic et al., 2010; Gjerdingen & Perrott, 2008; Schellenberg et al., 1999) suggest that the overall amount of listening one does in and of itself can shape auditory perception. Kliuchko and colleagues (2019), on the other hand, found that while jazz performers had developed stronger auditory-cortex discrimination for the sound features characteristic of jazz, this was not the case for listeners with a preference for jazz. Thus, devotees of the various genres we have studied who are not performers, but avid concert goers, dancers, and music consumers, as well as musicians with genre-specific training on the same instrument from different genres, would be useful participant pools. Investigating possible effects of genre-specific listening expertise and instrument practices on the perception and production of timing in the auditory domain are interesting topics for future research.

**Declarations**

*Funding*: This work was partially supported by the Research Council of Norway through its Centres of Excellence scheme, project number 262762, and the TIME project, grant number 249817.

*Conflicts of interest:* Not applicable.

*Ethics approval:* The study is approved by the Norwegian Centre for Research Data, NSD approval number 59513.

*Consent to participate:* Informed consent was obtained from all individual participants included in the study.

*Consent to publish:* All authors approved the manuscript and this submission.

*Open Practices Statement*: The datasets generated during the current study will be made available when the project is completed. None of the experiments was preregistered.

**Acknowledgments**

We want to thank Thea Paulsrud for assistance with preparing the manuscript.

**References**

Bengtsson, I. (1974). On notation of time, signature and rhythm in Swedish polskas. In G. Hilleström (Ed.), *Studia instrumentorum musicae popularis* *III* (pp. 22-31). Stockholm: Musikhistoriska museet/Nordiska Musikförlaget.

Bjerke, K. (2010). Timbral relationships and microrhythmic tension: shaping the groove experience through sound. In A. Danielsen (Ed.), *Musical Rhythm in the Age of Digital Reproduction*, (pp. 85-101). Farnham, UK: Ashgate/Routledge. <https://doi.org/10.4324/9781315596983-6>

Blom, J. P. (1981). The Dancing Fiddle: On the Expression of Rhythm in Hardingfele Slåtter. In

J. P. Blom, S. Nyhus & R. Sevåg (Eds.), *Norwegian Folk Music: Slåttar for the Harding*

*Fiddle, Vol. 7*, (pp. 305–312). Oslo: Universitetsforlaget.

Butler, M. J. (2006). *Unlocking the Groove: Rhythm, Meter, and Musical Design in Electronic Dance Music.* Bloomington: Indiana University Press.

Butterfield, M. W. (2010). Participatory discrepancies and the perception of beats in jazz. *Music Perception,* *27*(3), 157-176. <https://doi.org/10.1525/mp.2010.27.3.157>

Brøvig-Hanssen, R., Sandvik, B., Aareskjold-Drecker, J.M., & Danielsen, A. (2022). A Grid in Flux: Sound and Timing in Electronic Dance Music. *Music Theory Spectrum 44*(1) (in press)

Câmara, G. S. (2016). Swing in early Funk and Jazz-Funk (1967-1971): Micro-rhythmic and Macro-structural investigations. (Master thesis). Oslo: University of Oslo, Duo Research Archive. <http://urn.nb.no/URN:NBN:no-54627>

Cameron, D. J., & Grahn, J. A. (2014). Enhanced timing abilities in percussionists generalize to rhythms without a musical beat. *Frontiers in Human Neuroscience*, *8*, 1003. <https://doi.org/10.3389/fnhum.2014.01003>

Carlsen, K., & Witek, M. A. G. (2010). Simultaneous rhythmic events with different schematic affiliations: Microtiming and dynamic attending in two contemporary R&B grooves. In A. Danielsen (Ed.), *Musical Rhythm in the Age of Digital Reproduction* (pp. 51–68). Farnham, UK: Ashgate/Routledge.

Chen, J. L., Penhune, V. B., & Zatorre, R. J. (2008). Moving on time: brain network for auditory-motor synchronization is modulated by rhythm complexity and musical training. *Journal of cognitive neuroscience*, *20*(2), 226-239. <https://doi.org/10.1162/jocn.2008.20018>

Danielsen, A. (2006). *Presence and Pleasure: The Funk grooves of James Brown and Parliament.* Middletown, Connecticut: Wesleyan University Press.

Danielsen, A. (2010). Here, there, and everywhere. Three accounts of pulse in D'Angelo's 'Left and Right'. In A. Danielsen (Ed.), *Musical Rhythm in the Age of Digital Reproduction* (pp. 19-36). Farnham, UK: Ashgate/Routledge. <https://doi.org/10.4324/9781315596983>

Danielsen, Anne (2012). The Sound of Crossover: Micro-rhythm and Sonic Pleasure in Michael Jackson's "Don't Stop 'Til You Get Enough". *Popular Music and Society, 35*(2), 151-168. <https://doi.org/10.1080/03007766.2011.616298>

Danielsen, A., Haugen, M. R., & Jensenius, A. R. (2015). Moving to the beat: Studying entrainment to micro-rhythmic changes in pulse by motion capture. *Timing & Time Perception*, *3*(1–2), 133–154. <https://doi.org/10.1163/22134468-00002043>

Danielsen, Anne (2019). Pulse as Dynamic Attending: Analysing Beat Bin Metre in Neo Soul Grooves. In Ciro Scotto; Kenneth M. Smith & John Brackett (Eds.), The Routledge Companion to Popular Music Analysis: Expanding Approaches, (pp. 179-189). UK: Routledge. <https://doi.org/10.4324/9781315544700>

Danielsen, A., Nymoen, K., Anderson, E., Câmara, G. S., Langerød, M. T., Thompson, M. R., & London, J. (2019). Where is the beat in that note? Effects of attack, duration, and frequency on the perceived timing of musical and quasi-musical sounds. *Journal of Experimental Psychology: Human Perception and Performance*, *45*(3), 402-418. <https://doi.org/10.1037/xhp0000611>

Deutsch, D. (1991). The tritone paradox: An influence of language on music perception. *Music Perception, 8*(4), 335-347. <https://doi.org/10.2307/40285517>

Deutsch, D. (1997). The tritone paradox: A link between music and speech. *Current Directions in Psychological Science: a Journal of the American Psychological Society* *6(*6), 174-180. <https://doi.org/10.1111/1467-8721.ep10772951>

Ehrlé, N., & Samson, S. (2005). Auditory discrimination of anisochrony: Influence of the tempo and musical backgrounds of listeners. *Brain and Cognition*, *58*(1), 133-147. <https://doi.org/10.1016/j.bandc.2004.09.014>

Engel, A. K., Fries, P., & Singer, W. (2001). Dynamic predictions: oscillations and synchrony in top–down processing. *Nature Reviews Neuroscience*, *2*(10), 704-716. <https://doi.org/10.1038/35094565>

Faul, F., Erdfelder, E., Lang, A. G., & Buchner, A. (2007). G* Power 3: A flexible statistical power analysis program for the social, behavioral, and biomedical sciences. *Behavior research methods*, *39*(2), 175-191.

Filipic, S., Tillmann, B., & Bigand, E. (2010). Judging familiarity and emotion from very brief musical excerpts. *Psychonomic Bulletin & Review*, *17*(3), 335–341. <https://doi.org/10.3758/pbr.17.3.335>

Fujii, S., Hirashima, M., Kudo, K., Ohtsuki, T., Nakamura, Y., & Oda, S. (2011). Synchronization Error of Drum Kit Playing with a Metronome at Different Tempi by Professional Drummers. *Music Perception*, *28*(5), 491–503. <https://doi.org/10.1525/mp.2011.28.5.491>

Fujioka, T., Ross, B., Kakigi, R., Pantev, C., & Trainor, L. J. (2006). One year of musical training affects development of auditory cortical-evoked fields in young children. *Brain*, *129*(10), 2593-2608. <https://doi.org/10.1093/brain/awl247>

Gilbert, C. D., & Sigman, M. (2007). Brain states: top-down influences in sensory processing. *Neuron*, *54*(5), 677-696. https://doi.org/10.1016/j.neuron.2007.05.019

Gjerdingen, R. O., & Perrott, D. (2008). Scanning the Dial: The Rapid Recognition of Music Genres. *Journal of New Music Research*, *37*(2), 93–100. <https://doi.org/10.1080/09298210802479268>

Gordon, J. W. (1987). The perceptual attack time of musical tones. *The Journal of the Acoustical Society of America*, *82*(1), 88-105.

Grahn, J. A., & Brett, M. (2007). Rhythm and Beat Perception in Motor Areas of the Brain. *Journal of Cognitive Neuroscience*, *19*(5), 893–906. <https://doi.org/10.1162/jocn.2007.19.5.893>

Groven, E. (1971). Musikkstudiar – ikkje utgjevne før. [Music studies – not previously published.] 1. Rytmestudiar. [Rhythm studies.] In O. Fjalestad (Ed.), *Eivind Groven. Heiderskrift til 70-årsdagen 8. oktober 1971 [Eivind Groven. Festschrift to the 70th anniversary, October 8, 1971]* (pp. 93-102). Oslo: Noregs Boklag.

Hannon, E. E., Soley, G. & Ullal, S. (2012). Familiarity Overrides Complexity in Rhythm Perception: A Cross-Cultural Comparison of American and Turkish Listeners. *Journal of Experimental Psychology: Human Perception and Performance, 38*(3), 543–548. https://doi.org/10.1037/a0027225

Haugen, M. R. (2016). Investigating periodic body motions as a tacit reference structure in Norwegian telespringar performance. *Empirical Musicology Review*, *11*(3-4), 272-294. <https://doi.org/10.18061/emr.v11i3-4.5029>

Herholz, S. C., & Zatorre, R. J. (2012). Musical training as a framework for brain plasticity: behavior, function, and structure. *Neuron, 76*(3), 486-502. <https://doi.org/10.1016/j.neuron.2012.10.011>

Himberg, T. (2014). Interaction in Musical Time. (Doctoral dissertation). Apollo - University of Cambridge Repository. <https://doi.org/10.17863/CAM.15930>

Honing, H., & Ladinig, O. (2009). Exposure influences expressive timing judgments in music. *Journal of Experimental Psychology: Human Perception and Performance, 35*(1), 281–288. <https://doi.org/10.1037/a0012732>

Hove, M. J., Keller, P. E., & Krumhansl, C. L. (2007). Sensorimotor synchronization with chords containing tone-onset asynchronies. *Perception & Psychophysics*, *69*(5), 699-708.
<https://doi.org/10.3758/BF03193772>

Hu, A., Wang, M., Li, Y., Tang, Q. & Gu, F. (2020). Dong speakers outperform Mandarin speakers in behavioral pitch discrimination. *The Journal of the Acoustical Society of America*, *147*(1), EL62–EL65. <https://doi.org/10.1121/10.0000604>

Istók, E., Brattico, E., Jacobsen, T., Ritter, A., & Tervaniemi, M. (2013). ‘I love Rock ‘n’Roll’—Music genre preference modulates brain responses to music. *Biological psychology*, *92*(2), 142-151. <http://dx.doi.org/10.1016/j.biopsycho.2012.11.005>

Iversen, J. R., Patel, A. D., & Ohgushi, K. (2008). Perception of rhythmic grouping depends on auditory experience. *The Journal of the Acoustical Society of America*, *124*(4), 2263-2271. <https://doi.org/10.1121/1.2973189>

Jacoby, N., et. al. (2021). Universality and cross-cultural variation in mental represntations of music revealed by global comparisons of rhythm priors. Preprint: [https://psyarxiv.com/b879v](https://l.facebook.com/l.php?u=https%3A%2F%2Fpsyarxiv.com%2Fb879v%3Ffbclid%3DIwAR19zL6PGfVlRCmoBRygwKnA7z-drmBjrNza84ejrqi1ni4WPKioozxMywQ&h=AT1Z1-hBJoDMk6pAuxYtKBjdags9O8xuRvQ5t6kPOftWxNxV6sHwrDIRqjxKCb0QxekPAzAHFNPMAe2owcxm8yOxZu0EGdyCVzNTovlyLeCIedkHZUOMnqv87gcrxRGn3-ZltWc&__tn__=-UK-R&c%5b0%5d=AT3NHbOj3ZcNHiRKr1TxwoF-wfOji9_gOhCETOMbHfShsGL0sfSMTW1N4m5czoFDNCu8UzY1wMeEAWS05fPAcnMp6uhp-U18d_EXT4oQZKsZL1H4UN5DY8oALswrkeog-b9IhGqVgezKXLziNmOIoNyon3g)

Johansson, M. (2010a). *Rhythm into Style: Studying Asymmetrical Grooves in Norwegian Folk Music*. (PhD. thesis) Oslo: Department of Musicology, University of Oslo.

Johansson, M. (2010b). The Concept of Rhythmic Tolerance – Examining Flexible Grooves in Scandinavian Folk-fiddling. In A. Danielsen (Ed.), *Musical Rhythm in the Age of Digital Reproduction* (pp. 69-84). Farnham, Surrey, UK: Ashgate.

Johansson, M. (2017a). Empirical Research on Asymmetrical Rhythms in Scandinavian Folk Music: A Critical Review. *Studia Musicologica Norvegica,* *43*(1), 58-89. <https://doi.org/10.18261/issn.1504-2960-2017-01-05>

Johansson, M. (2017b). Non-isochronous Musical Meters: Towards a Multidimensional Model. *Ethnomusicology*, *61*(1): 31-51. <https://doi.org/10.5406/ethnomusicology.61.1.0031>

Jones, M. R., Jagacinski, R. J., Yee, W., Floyd, R. L., & Klapp, S. T. (1995). Tests of attentional flexibility in listening to polyrhythmic patterns. *Journal of Experimental Psychology: Human Perception and Performance*, 2*1*(2), 293–307. <https://doi.org/10.1037/0096-1523.21.2.293>

Jones, M. R., & Yee, W. (1997). Sensitivity to time change: The role of context and skill. *Journal of Experimental Psychology: Human Perception and Performance*, *23*(3), 693–709. <https://doi.org/10.1037/0096-1523.23.3.693>

Kliuchko, M., Brattico, E., Gold, B.P., Tervaniemi, M., Bogert, B., Toiviainen P., & Vuust, P. (2019) Fractionating auditory priors: A neural dissociation between active and passive experience of musical sounds. PLoS ONE 14(5): e0216499. <https://doi.org/10.1371/journal.pone.0216499>

Krause, V., Pollok, B., & Schnitzler, A. (2010). Perception in action: The impact of sensory information on sensorimotor synchronization in musicians and non-musicians. *Acta Psychologica, 133(*1), 28–37. <https://doi.org/10.1016/j.actpsy.2009.08.003>

Krumhansl, C. (2010). Plink: ‘Thin Slices’ of Music. *Music Perception,* *27*(5), 337-354. <https://doi.org/10.1525/mp.2010.27.5.337>

Kusumoto, K., & Moreton, E. (1997). Native language determines the parsing of nonlinguistic rhythmic stimuli. *The Journal of the Acoustical Society of America*, *102*(5), 3204–3204. <https://doi.org/10.1121/1.420936>

Kvifte, T. (2005). Fleksibelt metrum - noen refleksjoner med utgagnspunkt i norsk folkemusikk. In A. N. Ressem, E. H. Edvardsen, H.-H. Thedens, & R. Kvideland (Eds.), *Balladar & blue Hawaii* (pp. 199-206). Oslo: Novus forlag.

 Kvifte, T. (2007). Categories and timing: On the perception of meter. *Ethnomusicology, 51*(1), 64-84.

Large, E. W., & Jones, M. R. (1999). The dynamics of attending: How people track time-varying events. *Psychological review*, *106*(1), 119-159. <https://doi.org/10.1037/0033-295X.106.1.119>

Lartillot, O., and Toiviainen, P. (2007). A Matlab toolbox for musical feature extraction from audio. In S. Marchand (Ed.), *Proceedings of the 10th International Conference on Digital Audio Effects* (pp. 237–244). Bordeaux: University of Bordeaux 1.

Lartillot, O., Nymoen, K., Câmara, G. S., & Danielsen, A. (2021). Computational localization of attack regions through a direct observation of the audio waveform. *The Journal of the Acoustical Society of America*, *149*(1), 723-736. https://doi.org/10.1121/10.0003374

Leipold, S., Klein, C., & Jäncke, L. (2021). Musical expertise shapes functional and structural brain networks independent of absolute pitch ability. *Journal of Neuroscience*, *41*(11), 2496-2511.

London, J., Nymoen, K., Langerød, M. T., Thompson, M. R., Code, D. L., & Danielsen, A. (2019). A comparison of methods for investigating the perceptual center of musical sounds. *Attention, Perception, & Psychophysics*, *81*(6), 2088-2101. <https://doi.org/10.3758/s13414-019-01747-y>

Manning, F. C. & Schutz, M. (2015). Trained to keep a beat: movement-related enhancements to timing perception in percussionists and non-percussionists. *Psychological Research, 80*(4), 532–542. <https://doi.org/10.1007/s00426-015-0678-5>

Manning, F. C., Harris, J. & Schutz, M. (2017). Temporal prediction abilities are mediated by motor effector and rhythmic expertise. *Experimental Brain Research, 235*(3), 861–871. <https://doi.org/10.1007/s00221-016-4845-8>

Manning, F. C., Siminoski, A., & Schutz, M. (2020). Exploring the Effects of Effectors: Finger Synchronization Aids Rhythm Perception Similarly in Both Pianists and Non-pianists. *Music Perception*, *37*(3), 196-207. <https://doi.org/10.1525/mp.2020.37.3.196>

Margulis, E. H., Mlsna, L. M., Uppunda, A. K., Parrish, T. B., & Wong, P. C. M. (2009). Selective neurophysiologic responses to music in instrumentalists with different listening biographies. *Human Brain Mapping*, *30*(1), 267–275. <https://doi.org/10.1002/hbm.20503>

Matthews, T. E., Thibodeau, J. N. L., Gunther, B. P., & Penhune, V. B. (2016). The Impact of Instrument-Specific Musical Training on Rhythm Perception and Production. *Frontiers in Psychology*, *7*, 69. <https://doi.org/10.3389/fpsyg.2016.00069>

Monson, I. (1996). *Saying something: jazz improvisation and interaction*. Chicago, IL: University of Chicago Press.

Morton, J., Marcus, S., & Frankish, C. (1976). Perceptual centers (P-centers). *Psychological Review*, *83*(5), 405-408. <http://dx.doi.org/10.1037/0033-295X.83.5.405>

Pantev, C., Roberts, L. E., Schulz, M., Engelien, A., & Ross, B. (2001a). Timbre-specific enhancement of auditory cortical representations in musicians. *Neuroreport*, *12*(1), 169-174. <https://doi.org/10.1097/00001756-200101220-00041>

Pantev, C., Engelien, A., Candia, V., & Elbert, T. (2001b). Representational cortex in musicians: plastic alterations in response to musical practice. *Annals of the New York Academy of Sciences*, *930*(1), 300-314.

Patel, A. D., Iversen, J. R., & Ohgushi, K. (2006). Nonlinguistic rhythm perception depends on culture and reflects the rhythms of speech: Evidence from English and Japanese. *The Journal of the Acoustical Society of America*, *120*(5), 3167–3167. <https://doi.org/10.1121/1.4787902>

Polak, R., Jacoby, N., Fischinger, T., Goldberg, D., Holzapfel, A., & London, J. (2018). Rhythmic Prototypes Across Cultures. *Music Perception*, *36*(1), 1–23. <https://doi.org/10.1525/mp.2018.36.1.1>

Rammsayer, T., & Altenmüller, E. (2006). Temporal Information Processing in Musicians and Nonmusicians. *Music Perception*, *24*(1), 37–48. <https://doi.org/10.1525/mp.2006.24.1.37>

Repp, B. H. (2010). Sensorimotor synchronization and perception of timing: Effects of music training and task experience. *Human Movement Science*, *29*(2), 200–213. <https://doi.org/10.1016/j.humov.2009.08.002>

Repp, B. H., & Doggett, R. (2007). Tapping to a very slow beat: A comparison of musicians and non-musicians. *Music Perception, 24*(4), 367–376. <https://doi.org/10.1525/MP.2007.24.4.367>

Repp, B. H., & Su, Y.-H. (2013). Sensorimotor synchronization: A review of recent research (2006–2012). *Psychonomic Bulletin & Review*, *20*(3), 403–452. <https://doi.org/10.3758/s13423-012-0371-2>

Rüsseler, J., Altenmüller, E., Nager, W., Kohlmetz, C., & Münte, T. F. (2001). Event-related brain potentials to sound omissions differ in musicians and non-musicians. *Neuroscience letters*, *308*(1), 33-36. <https://doi.org/10.1016/S0304-3940(01)01977-2>

Schellenberg, E. G., Iverson, P., & Mckinnon, M. C. (1999). Name that tune: Identifying popular recordings from brief excerpts. *Psychonomic Bulletin & Review*, *6*(4), 641–646. <https://doi.org/10.3758/bf03212973>

Senn, O., Kilchenmann, L., Bechtold, T., & Hoesl, F. (2018). Groove in drum patterns as a function of both rhythmic properties and listeners' attitudes. *PLoS ONE, 13*(6), e0199604. https://doi.org/10.1371/journal.pone.0199604

Skaansar, J. F., Laeng, B., & Danielsen, A. (2019). Microtiming and mental effort: Onset asynchronies in musical rhythm modulate pupil size. *Music Perception*, *37*(2), 111-133. <https://doi-org.ezproxy.uio.no/10.1525/mp.2019.37.2.111>

Teki, S., Grube, M., Kumar, S., & Griffiths, T. D. (2011). Distinct neural substrates of duration-based and beat-based auditory timing. *Journal of Neuroscience*, *31*(10), 3805-3812. <https://doi.org/10.1523/JNEUROSCI.5561-10.2011>

Ullal-Gupta, S., Hannon, E. E. & Snyder, J. S. (2014). Tapping to a Slow Tempo in the Presence of Simple and Complex Meters Reveals Experience-Specific Biases for Processing Music. *PLoS ONE, 9*(7), e102962. https://doi.org/10.1371/journal.pone.0102962

Van Vugt, F. T., & Tillmann, B. (2014). Thresholds of auditory-motor coupling measured with a simple task in musicians and non-musicians: was the sound simultaneous to the key press? *PloS One*, *9*(2), e87176. <https://doi.org/10.1371/journal.pone.0087176>

Villing, R. (2010). Hearing the moment: Measures and models of the perceptual centre. (Doctoral dissertation, National University of Ireland Maynooth).

Vos, J., & Rasch, R. (1981). The perceptual onset of musical tones. *Perception & psychophysics*, *29*(4), 323-335.

Vuust, P., Brattico, E., Seppänen, M., Näätänen, R. & Tervaniemi, M. (2012). The sound of music: Differentiating musicians using a fast, musical multi-feature mismatch negativity paradigm. *Neuropsychologia*, 50(7), 1432–1443. <https://doi.org/10.1016/j.neuropsychologia.2012.02.028>

Walton, J. P. (2010). Timing is everything: temporal processing deficits in the aged auditory brainstem. *Hearing research*, *264*(1-2), 63-69. <https://doi.org/10.1016/j.heares.2010.03.002>

Wright, M. J. (2008). The shape of an instant: measuring and modeling perceptual attack time with probability density. (Doctoral dissertation, Department of Music, Stanford University).

Yee, W., Holleran, S., & Jones, M. R. (1994). Sensitivity to event timing in regular and irregular sequences: Influences of musical skill. *Perception & Psychophysics,* *56*(4), 461–471. <https://doi.org/10.3758/bf03206737>

Zatorre, R. J., Chen, J. L., & Penhune, V. B. (2007). When the brain plays music: auditory–motor interactions in music perception and production. *Nature Reviews Neuroscience, 8*(7), 547–558. <https://doi.org/10.1038/nrn2152>

**Supplemental Materials**

**Table A1.** Results from the background questionnaire.

|  | | **Overall (N=59)** | | **Folk (N=19)** | | | **Jazz (N=20)** | | | | **Producer (N=20)** | | |
| --- | --- | --- | --- | --- | --- | --- | --- | --- | --- | --- | --- | --- | --- |
| **Mean Age** | | 34,7 | | 40,5 | | | 38,3 | | | | 27,3 | | |
| **Sex (%female)** | | 29% | | 42% | | | 25% | | | | 20% | | |
| **Start age main genre*** | | | | |  | | | |  | | |  |  |
| 0-10 | 27% | | 32% | | | 20% | | | | 30% | | |  |
| 10-15 | 36% | | 26% | | | 30% | | | | 50% | | |  |
| 15-20 / adult | 36% | | 37% | | | 50% | | | | 20% | | |  |
| **Instrument training in second genre (share of group)** | | | | | | | | | | | | |  |
| Folk |  | |  | | |  | | | |  | | |  |
| Jazz |  | | 11% | | |  | | | | 20% | | |  |
| EDM/Hip-hop |  | | 5% | | | 10% | | | |  | | |  |
| Pop/Rock |  | | 11% | | | 70% | | | | 35% | | |  |
| Classical |  | | 5% | | | 15% | | | | 5% | | |  |
| Other |  | | 11% | | |  | | | |  | | |  |
| **Type of instrument training and production experience (share of group)** | | | | | | | | | | | | |  |
| Bow/Breath | 39% | | 58% | | | 30% | | | | 30% | | |  |
| Tap/Pluck | 54% | | 42% | | | 70% | | | | 50% | | |  |
| Production |  | | 5% | | | 60% | | | | 90% | | |  |
| **Current practice*** | | | | | | | |  |  |  |  |  |  |
| 1-2 h/ week | 5% | | - | | | 10% | | | | 15% | | |  |
| 2-6 h/ week | 34% | | 63% | | | 5% | | | | 35% | | |  |
| 6 + h/ week | 51% | | 32% | | | 85% | | | | 35% | | |  |
| **Listening to music** | | | | |  | | | |  | | |  |  |
| 1-5 h/week | 27% | | 53% | | | 25% | | | | 5% | | |  |
| 1-2 h/day | 25% | | 16% | | | 30% | | | | 30% | | |  |
| 2-5 h/day | 25% | | 11% | | | 30% | | | | 35% | | |  |
| 5+ h/ day | 22% | | 16% | | | 15% | | | | 30% | | |  |

** Missing percentages in the Folk group are due to missing answers.*

*Abbreviations: Bow/Breath = bowed string instruments, voice, wind instruments. Tap/Pluck = drums, plectrum-played string instruments, piano/electric piano.*

**Table A2.** Sound stimuli - Musical sounds (upper panel) and Quasi-musical sounds (lower panel). Measures of the waveform obtained using Amadeus Pro, version 2.4.5 (HairerSoft).

|  | **Click** | **Fast**  **Short** | **Fast**  **Long** | **Slow Short** | **Slow**  **Long** | **Fast**  **Short** | **Fast**  **Long** | **Slow**  **Short** | **Slow Long** |
| --- | --- | --- | --- | --- | --- | --- | --- | --- | --- |
| **Sound** |  | **Electronic** | **Electronic** | **Electronic** | **Electronic** | **Organic** | **Organic** | **Organic** | **Organic** |
| **Instrument** |  | 808  Kick drum | Synth bass | Synth bass | Synth bass | Acoustic kick drum | El Bass | Fiddle | Fiddle |
| **Attack** | 0 ms | Fast | Fast | Slow | Slow | Fast | Fast | Slow | Slow |
| **Rise Time**  (from onset to energy peak) |  | 1 ms | 1 ms | ≈ 74 ms | ≈ 122 ms | 13 ms | 22 ms | ≈ 168 ms | ≈ 226 ms |
| **Duration**  (manual /  Lartillot et al. 2021) | 1  / 1 | ≈ 100*  / 238 | 520  / 519 | 210  / 208 | 530  / 534 | ≈ 100*  / 180 | 490  / 493 | 300  / 306 | 580  / 589 |
| **Pitch** (Hz, where relevant) |  |  | c^-1^  (65.4) | c^-1^  (65.4) | c^-1^  (65.4) |  | a^-2^  (55.0) | F^1^  (349.2)  (+ D^1^ )** | F^1^  (349.2)  (+ D^1^ )** |
| **Spectral Centroid**  (Hz, MIR toolbox) | 3531.5 | 313.8 | 172.7 | 313.0 | 297.8 | 581.4 | 406.2 | 2316.8 | 2405.0 |

**Impulsive, percussive sound. **Resonating sympathetic string on the fiddle.*

*Abbreviations: Fast = fast, impulsive attack, Slow = slow, gradual attack, Short = short duration, Long = long duration.*

|  | **Fast**  **Short** | **Fast**  **Long** | **Slow**  **Short** | **Slow**  **Long** |
| --- | --- | --- | --- | --- |
| **Rise Time** | 3 ms | 3 ms | 50 ms | 50 ms |
| **Duration** | 100 ms | 400 ms | 100 ms | 400 ms |
| **Center Frequency** | 100 Hz | 100 Hz | 100 Hz | 100 Hz |

*Abbreviations: Fast = fast, impulsive attack, Slow = slow, gradual attack, Short = short duration, Long = long duration.*

**Table A3.** Mean P-center and mean P-center variability per stimulus for click alignment and tapping trials for each group.

|  | Folk_CLICK | Jazz_CLICK | Prod_CLICK | Folk_TAP | Jazz_TAP | Prod_TAP |  |
| --- | --- | --- | --- | --- | --- | --- | --- |
| *P-center (in milliseconds relative to stimulus onset)* | | | | | | | |
| Click | 3 | -1 | 1 | -25 | -11 | -18 |  |
| Acoustic kick (fast-short) | 11 | 12 | 10 | -4 | 3 | 2 |  |
| El.bass (fast-long) | 37 | 36 | 29 | 26 | 31 | 29 |  |
| Fiddle (slow-short) | 40 | 41 | 26 | 47 | 34 | 26 |  |
| Fiddle (slow-long) | 233 | 185 | 90 | 189 | 144 | 66 |  |
| 808 kick (fast-short) | -3 | 2 | -1 | -16 | -9 | -11 |  |
| Synthbass (fast-long) | 6 | 6 | 1 | -9 | -1 | -2 |  |
| Synthbass (slow-short) | 55 | 64 | 57 | 41 | 45 | 45 |  |
| Synthbass (slow-long) | 127 | 109 | 102 | 94 | 91 | 86 |  |
| Noise (fast-short) | 0 | 5 | 4 | -12 | -2 | -2 |  |
| Noise (fast-long) | 4 | 9 | 5 | 10 | 7 | 6 |  |
| Noise (slow-short) | 17 | 12 | 14 | 1 | 12 | 8 |  |
| Noise (slow-long) | 27 | 22 | 22 | 27 | 23 | 20 |  |
| *P-center variability (in milliseconds)* | | | | | | | |
| Click | 4 | 2 | 2 | 18 | 13 | 18 |  |
| Acoustic kick (fast-short) | 15 | 9 | 10 | 15 | 9 | 11 |  |
| El.bass (fast-long) | 18 | 16 | 12 | 16 | 14 | 12 |  |
| Fiddle (slow-short) | 28 | 29 | 17 | 18 | 14 | 16 |  |
| Fiddle (slow-long) | 66 | 69 | 37 | 40 | 33 | 22 |  |
| 808 kick (fast-short) | 11 | 6 | 6 | 17 | 15 | 13 |  |
| Synthbass (fast-long) | 20 | 11 | 10 | 16 | 11 | 13 |  |
| Synthbass (slow-short) | 28 | 19 | 17 | 17 | 12 | 13 |  |
| Synthbass (slow-long) | 26 | 28 | 23 | 18 | 15 | 20 |  |
| Noise (fast-short) | 16 | 14 | 11 | 15 | 13 | 12 |  |
| Noise (fast-long) | 20 | 14 | 11 | 16 | 12 | 13 |  |
| Noise (slow-short) | 26 | 19 | 16 | 18 | 12 | 13 |  |
| Noise (slow-long) | 27 | 17 | 19 | 14 | 13 | 13 |  |

*Abbreviations: Folk=folk musicians, Jazz=jazz musicians, Prod=producers, CLICK = click alignment, TAP = tapping.*

1. Spectral centroid was calculated using MIR toolbox version 1.7.2 for Matlab (Lartillot and Toiviainen, 2007), duration was calculated as the time difference between offset and onset, using the waveform-based offset/onset detection method elaborated in Lartillot et. al., (2021) (MIR toolbox version 1.8). [↑](#footnote-ref-1)
